# Three-dimensional tumor cell growth stimulates autophagic flux and recapitulates chemotherapy resistance

**DOI:** 10.1038/cddis.2017.398

**Published:** 2017-08-24

**Authors:** Corinna Bingel, Emily Koeneke, Johannes Ridinger, Annika Bittmann, Martin Sill, Heike Peterziel, Jagoda K Wrobel, Inga Rettig, Till Milde, Uta Fernekorn, Frank Weise, Andreas Schober, Olaf Witt, Ina Oehme

**Affiliations:** 1 Clinical Cooperation Unit Pediatric Oncology, German Cancer Research Center (DKFZ), INF 280, D-69120 Heidelberg, Germany; 2 Translational Program, Hopp Children’s Cancer Center at NCT Heidelberg (KiTZ), Heidelberg, Germany; 3 Division of Biostatistics, German Cancer Research Center (DKFZ), Heidelberg, Germany; 4 Center for Individualized Pediatric Oncology (ZIPO) and Brain Tumors, Department of Pediatric Oncology, Hematology and Immunology, University Hospital Heidelberg, Heidelberg, Germany; 5 Department of Nano-Biosystem Technology, Technische Universität Ilmenau, Ilmenau, Germany

## Abstract

Current preclinical models in tumor biology are limited in their ability to recapitulate relevant (patho-) physiological processes, including autophagy. Three-dimensional (3D) growth cultures have frequently been proposed to overcome the lack of correlation between two-dimensional (2D) monolayer cell cultures and human tumors in preclinical drug testing. Besides 3D growth, it is also advantageous to simulate shear stress, compound flux and removal of metabolites, e.g., via bioreactor systems, through which culture medium is constantly pumped at a flow rate reflecting physiological conditions. Here we show that both static 3D growth and 3D growth within a bioreactor system modulate key hallmarks of cancer cells, including proliferation and cell death as well as macroautophagy, a recycling pathway often activated by highly proliferative tumors to cope with metabolic stress. The autophagy-related gene expression profiles of 2D-grown cells are substantially different from those of 3D-grown cells and tumor tissue. Autophagy-controlling transcription factors, such as TFEB and FOXO3, are upregulated in tumors, and 3D-grown cells have increased expression compared with cells grown in 2D conditions. Three-dimensional cultures depleted of the autophagy mediators BECN1, ATG5 or ATG7 or the transcription factor FOXO3, are more sensitive to cytotoxic treatment. Accordingly, combining cytotoxic treatment with compounds affecting late autophagic flux, such as chloroquine, renders the 3D-grown cells more susceptible to therapy. Altogether, 3D cultures are a valuable tool to study drug response of tumor cells, as these models more closely mimic tumor (patho-)physiology, including the upregulation of tumor relevant pathways, such as autophagy.

The success rates for investigational cancer drugs in clinical development are poor. The clinical approval rate of compounds for the treatment of solid tumors is 10% or less.^1, 2^ Improving basic research models is critical for achieving clinical success. Conventionally, preclinical assessment of chemotherapeutic effectiveness starts in two-dimensional (2D) cultures, where cell-cell contacts, cell shape and morphology significantly differ from tumor cells in a physiological setting. All of these features strongly influence cellular growth, behavior and metabolism.^3^ Three-dimensional (3D) growth cultures have been introduced for preclinical drug screening to improve the correlation between cell cultures and tumors.^4^ Three-dimensional cell growth is associated with a spherical shape, affecting gene and protein expression, survival, proliferation, differentiation, and metabolism.^5, 6^ Furthermore, 3D-grown tumor cells display enhanced resistance to radio- and chemotherapy.^7, 8^ Additional important characteristics of the physiological setting include the flow of extracellular fluids, leading to shear stress, compound flux and removal of metabolites. Small 3D bioreactor systems mimic these properties by pumping medium at a physiologically representative flow rate.^6, 9^

Neuroblastoma, a common pediatric tumor of the sympathetic nervous system, is characterized by a wide range of clinical courses.^10^ Despite intensification of treatment, high-risk neuroblastoma patients have a very poor prognosis due to chemotherapy resistance.^10, 11^ We and others have previously reported that macroautophagy (hereafter autophagy) supports chemotherapy resistance in neuroblastoma cells.^12, 13^ Thus, neuroblastoma is a good model to investigate autophagy-related drug resistance.

Autophagy is an evolutionarily conserved process, involving sequestration of cytoplasmic components within a double-membrane structure (autophagosome) and subsequent delivery to lysosomes for degradation.^14^ Metabolic or therapeutic stress, e.g. DNA-damaging drugs, may induce autophagy,^15^ which is regulated by autophagy-related (*ATG*) genes.^14^ Whereas autophagy plays a tumor-suppressive role in early stages of tumorigenesis, it can support growth at later stages, allowing tumor cells to survive with limited oxygen and nutrients, as well as under cytotoxic treatment conditions.^16^

Histone deacetylase (HDAC) inhibitors interfere with key tumor-relevant pathways, including proliferation, apoptosis, differentiation and autophagy in several cancer entities *in vitro* and *in vivo.*^17, 18, 19^ The human HDAC family comprises four classes: class I, II, subdivided into classes IIa and IIb, III and IV.^20, 21, 22^ Class IIb family members (HDACs 6 and 10) are linked to cellular stress, protein degradation and autophagy.^12, 23, 24, 25, 26^ We have previously identified HDAC10 as a mediator of autophagic flux in neuroblastoma and inhibition as well as depletion of HDAC10 sensitized monolayer neuroblastoma cells to cytotoxic drugs.^12^

It remains unclear how different cell culture settings influence autophagy, particularly in the context of drug development and therapy resistance. Here we show that 3D culture models are a suitable tool for screening drug responses of tumors. Additionally, these models are useful for mechanistically studying cancer-relevant processes, as they more accurately resemble transcriptional profiles of tumors and recapitulate the regulation of physiologically relevant pathways, including autophagy. Our data suggest that 3D tumor models are advantageous when studying autophagy-targeting treatment and resistance mechanisms.

## Results

### Expression profiles from *MYCN*-amplified neuroblastoma cell lines and tissues are highly discrepant

**Figure 1:**
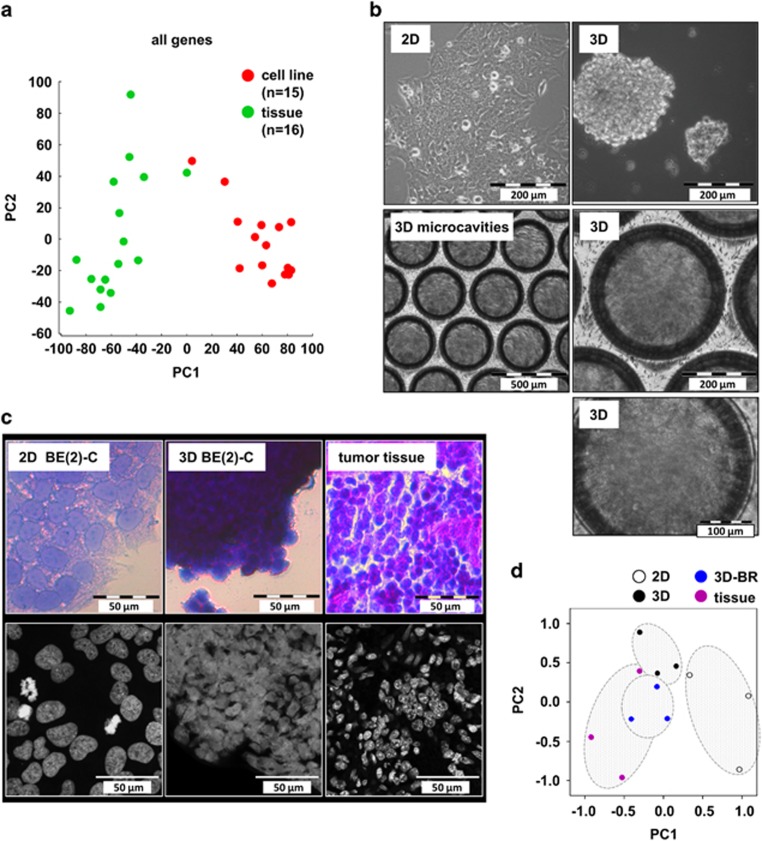
Three-dimensional cell culture models recapitulate typical features of neuroblastoma tumors. (**a**) Principal component analysis (PCA) of transcriptomes, using gene expression profiles from 15 *MYCN* amplified neuroblastoma cell lines (red) and 16 *MYCN* amplified neuroblastoma tissue samples (green) via the web-based R2 microarray database (http://r2.amc.nl) and the data set of mixed neuroblastoma cell lines/tissues (‘Versteeg’). The first two principal components, PC1 and PC2, are shown. (**b**) BE(2)-C cells grown as monolayers under normal 2D conditions and in 3D culture on collagen-coated polymeric chips with multiple microcavities. Upper left: monolayer, scale bar: 200 *μ*m. Upper right: Spheroidal 3D-cultured cell cluster freed from its chip, scale bar: 200 *μ*m. Middle panel: 3D culture on chip. Microcavities filled with cells are shown with lower (scale bar: 500 *μ*m) and higher (scale bar: 200 *μ*m) magnification. Lower panel: highest magnification (scale bar: 100 *μ*m). (**c**) Representative images of HE (upper panel) and nuclear staining (lower panel) of BE(2)-C cells grown under 2D (left) or 3D conditions (middle), and neuroblastoma tissue (right). Cells grown for 72 h under 3D conditions were freed from the polymeric chip for staining. Note that 3D culture leads to a small round blue cell morphology whereas 2D cultures are settled on a flat surface and develop a more ganglionic phenotype. In addition, under 3D culture conditions, the nuclei are reduced in size. (**d**) Principal component analysis (PCA) of transcriptomes from 2D (open), 3D-static (3D; black), 3D bioreactor (3D-BR; blue) 6 d cultures of BE(2)-C cells and three neuroblastoma patient samples (tissue; magenta). The first two principal components PC1 and PC2 are shown. Note that PC1 separates the transcriptomes into two clusters: Cluster one, in the negative range of the *x*axis includes neuroblastoma tissue samples and both kinds of 3D-cultured cells and a separate cluster (positive range) that contains transcriptomes of the 2D-cultured cells

Tumor cells tend to lose many hallmarks of cancer over time when cultured *in vitro* under standard conditions. We explored differences between cultured tumor cells and primary tumor tissues by comparing the gene expression profiles of *MYCN* amplified neuroblastoma cell lines with tissue samples from a publically available data set (Mixed Neuroblastoma – Versteeg; R2 database). A principal component analysis (PCA) performed with all genes revealed two distinct clusters (Figure 1a, Supplementary Figure S1a), indicating that the gene expression profile after two-dimensional (2D) growth shifts away from tumor tissue. We hypothesized that three-dimensional (3D) growth would better recapitulate neuroblastoma physiology. Thus, we seeded neuroblastoma cells in a collagen type I-coated, ridged scaffold model, which yielded reproducible one-size 3D structures. The polymeric scaffolds contain 187 microcavities per chip, promoting 3D growth of multicellular spheroids approximately 200 *μ*m in diameter (Figure 1b). Corroborating previous findings,^27^ the collagen substratum in our system was a suitable extracellular matrix protein, affecting neither population doubling time nor response to cytotoxic treatment (Supplementary Table 1). To account for additional physiological factors, we also inserted the chips into a bioreactor system,^9^ which simulates the flow of extracellular fluids (Supplementary Figures S1b and c).

A typical histomorphological feature of undifferentiated and aggressive neuroblastoma tumors is its small round blue cell phenotype (Supplementary Figure S1d), which is lost upon growth under classical 2D culture conditions. Two-dimensionally grown cells exhibit flat morphology with much larger nuclei and fewer cell–cell contacts. In contrast, 3D-grown cells more closely resembled small round blue cells, characteristic for undifferentiated neuroblastoma tissue (Figure 1c, Supplementary Figure S1e). Solid tumors are also metabolically heterogeneous, with areas of maximal growth, slow-growing starved regions or even necrotic parts.^28^ Similar structures are observable within multicellular spheroid 3D models.^29^ We investigated whether growing neuroblastoma cells in the 3D-chip model affected overall cell proliferation and death rate. Three-dimensionally grown cells exhibited an increased population doubling time, but still grew exponentially, with slightly decreased overall viability (Supplementary Table 2). To investigate whether 3D growth affects gene transcription, we analyzed the gene expression patterns of BE(2)-C cells grown in 2D, 3D and bioreactor-3D cultures and compared them to tumor tissue samples from three different neuroblastoma patients (German Neuroblastoma Trial cohort^30^) resembling the characteristics of BE(2)-C cells (stage 4, amplified *MYCN*, undifferentiated and 1p deletion). A PCA revealed that 3D-cultured (static and bioreactor) neuroblastoma transcriptomes were closer to the neuroblastoma tissue profile than the 2D samples (Figure 1d, Supplementary Figure S1f). These results support our hypothesis that 3D growth of cells not only resembles more phenotypic histology, but also reduces the discrepancy between transcriptional programs in cultured cells and primary tumor material.

### Resistance to cytotoxic treatment is enhanced in 3D-grown neuroblastoma cells

**Figure 2:**
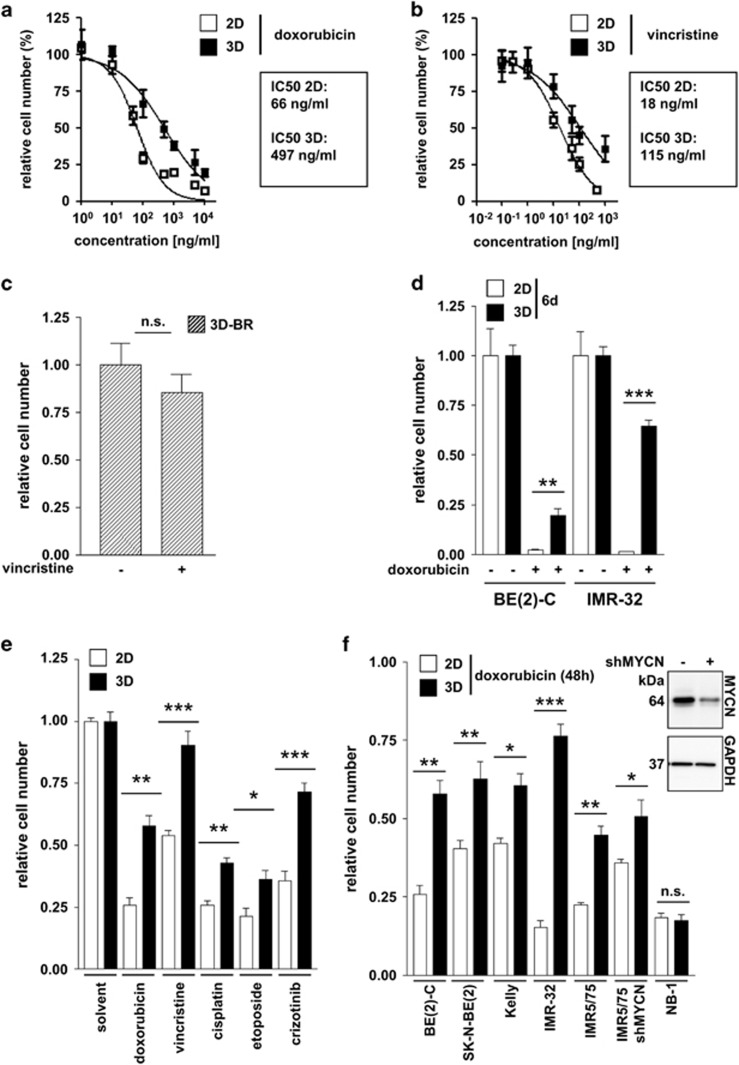
Three-dimensional cell growth affects drug responsiveness of neuroblastoma cells. BE(2)-C cells grown in monolayer (2D) or in 3D were treated 24 h post-seeding with doxorubicin (**a**) or vincristine (**b**) in various concentrations for 48 h. IC_50_ values were calculated with GraphPad Prism (San Diego, CA, USA) (function: log(inhibitor) *versus* normalized response; variable slope). (**c**) One day after seeding, chips containing 3D-cultured BE(2)-C cells were transferred into the bioreactor system (3D-BR) and medium or medium containing vincristine (10 ng/ml) was pumped through the system for 48 h. (**d**) One day after seeding, 2D- and 3D-cultured BE(2)-C or IMR-32 cells were treated for 6 d with doxorubicin, where indicated. Doxorubicin concentrations: BE(2)-C: 0.5 *μ*g/ml and IMR-32: 0.01 *μ*g/ml. (**e**) BE(2)-C cells grown in 2D or 3D were treated 24 h post-seeding with doxorubicin (0.5 *μ*g/ml), vincristine (0.01 *μ*g/ml), cisplatin (15 *μ*g/ml), etoposide (7.5 *μ*g/ml) or crizotinib (0.8 *μ*M). (**f**) A panel of neuroblastoma cell lines (BE(2)-C, SK-N-BE, Kelly, IMR-32, tetracycline-inducible *shMYCN* IMR5/75 (on/off) and NB1) grown in monolayer (2D) or in 3D was treated 24 h post-seeding with doxorubicin for 48 h. Doxorubicin concentrations: BE(2)-C: 0.5 *μ*g/ml; SK-N-BE: 0.1 *μ*g/ml, Kelly: 0.05 *μ*g/ml, IMR-32: 0.01 *μ*g/ml, IMR5/75: 0.01 *μ*g/ml, NB1: 0.5 *μ*g/ml. Inlay: Western blot displaying MYCN expression with (+) or without (−) doxycycline treatment of inducible *shMYCN* IMR5/75 cells. (**a**–**f**) Number of viable cells was assessed by automated cell counting and trypan blue exclusion and was normalized to untreated control of each condition. Significant differences between groups were tested using an unpaired, two-tailed *t*-test. n.s., not significant; * *P*<0.05; **P<0.01; ***P<0.001

To compare drug response, we treated 2D- and 3D-grown neuroblastoma cells with the cytotoxic compounds doxorubicin and vincristine. Three-dimensional growth strongly hampered the drug-induced reduction in BE(2)-C cell number compared to 2D growth, with the IC50 values for doxorubicin and vincristine increasing approximately 7- and 5-fold, respectively (Figures 2a and b). IMR-32 cells were similarly affected (Supplementary Figures S2a and b). Neuroblastoma cells grown three-dimensionally in the bioreactor system were completely resistant to vincristine (Figure 2c). To examine long-term effects of drug treatment, we treated 2D- and 3D-cultured BE(2)-C as well as IMR-32 cells for six days with doxorubicin and confirmed prolonged resistance in tumor cells cultured under 3D conditions (Figure 2d). Using the 3D-BE(2)-C model we tested other clinically-relevant drugs, such as cisplatin, etoposide and the MET/ALK-inhibitor crizotinib. In all cases, 3D-cultured cells exhibited a weaker response than 2D-grown cells (Figure 2e). Moreover, six out of a panel of seven neuroblastoma cell lines had more cells survive doxorubicin treatment under 3D compared with 2D conditions (Figure 2f). Only NB-1 cells were highly sensitive to doxorubicin, irrespective of culture conditions. Both *MYCN* amplified and MYCN-depleted (shMYCN) IMR5/75 cells respond less to doxorubicin when cultured in 3D. However, the difference in drug sensitivity between both conditions was greater among cells expressing MYCN (2-fold) compared to MYCN-depleted cells (1.4-fold), suggesting MYCN involvement in mediating resistance. Our findings support the notion that the lower sensitivity to cytotoxic drugs observed in 3D cultures more accurately recapitulates chemotherapy resistance than 2D-grown cells and that more representative culture conditions could avoid a misleading prediction of drug sensitivity.

### Drug resistance of three-dimensionally grown tumor cells translates into a suppressed apoptotic response

**Figure 3:**
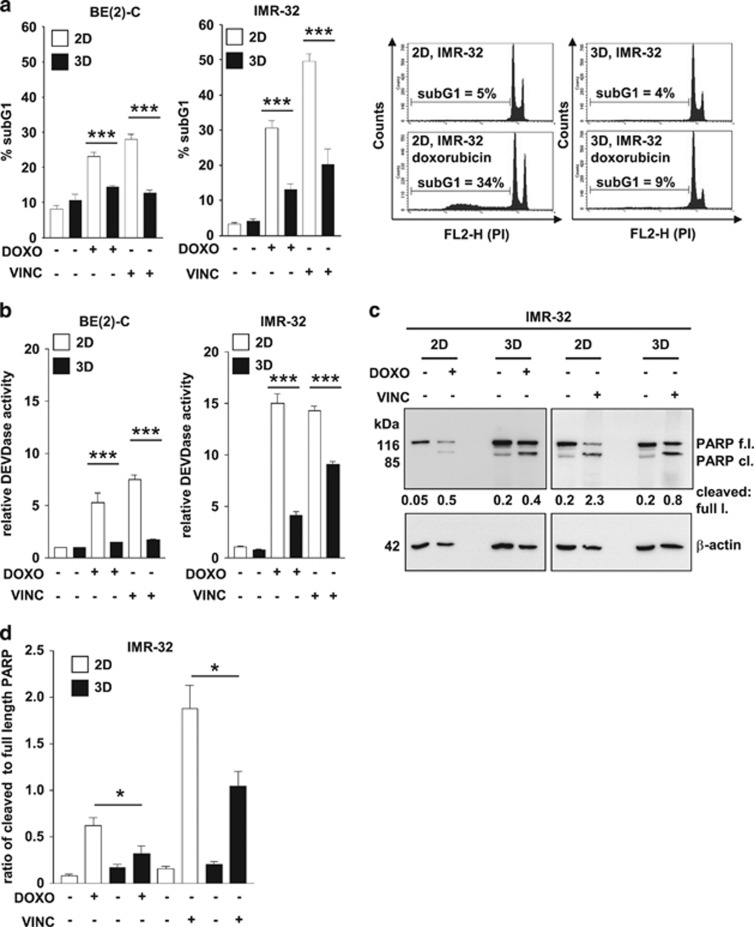
Three-dimensional cell growth impairs apoptosis in neuroblastoma cells. (**a**) BE(2)-C and IMR-32 cells were grown under 2D- and 3D conditions for 96 h. After 24 h, doxorubicin or vincristine was added to the media, and 72 h later, the cells and supernatants were collected for the detection of cell death by propidium iodide (PI) staining of ethanol-fixed cells. Representative histograms for solvent control and doxorubicin-treated IMR-32 cells are shown on the right. (**b**) One day after seeding, 2D- and 3D-cultured BE(2)-C and IMR-32 cells were treated for another 48 h with doxorubicin or vincristine, where indicated. Caspase-3 (DEVDase) activity was measured using a fluorometric assay with activity (slope/min) measured relative to untreated 2D cells. (**c**) Western blot analysis of PARP cleavage in 2D- and 3D-grown IMR-32 cells 48 h after treatment with doxorubicin or vincristine where indicated. Cells were cultured for 72 h in total. *β*-Actin served as a loading control. Numbers indicate the ratio of cleaved to full-length PARP. f.l., full-length. (**d**) Quantification of three biological replicates of the detection of PARP cleavage in 2D- and 3D-grown IMR-32 cells 48 h after treatment with doxorubicin or vincristine where indicated. Cells were cultured for 72 h in total. (**a**,**b** and **d**) Bars represent mean values of at least three independent experiments, error bars represent S.E.M. Significant differences between groups were tested using an unpaired, two-tailed *t*-test. *P<0.05, ***P<0.001. (**a**–**d**) Treatment of BE(2)-C: 0.5 *μ*g/ml doxorubicin or 10 ng/ml vincristine. Treatment of IMR-32: 0.01 *μ*g/ml doxorubicin or 3 ng/ml vincristine

As 3D growth yields more treatment-surviving neuroblastoma cells, we investigated whether programmed cell death was affected. The number of dead cells (BE(2)-C, IMR-32) in the subG1-area of the cell cycle profile following doxorubicin or vincristine treatment clearly differed between 2D and 3D conditions (Figure 3a). Correspondingly, significantly reduced activation of effector caspases in response to drug treatment under 3D culture conditions was observed in both cell lines (Figure 3b). The difference in caspase activity in 3D *versus* 2D-grown cells was also reflected by different levels of PARP cleavage, exemplarily shown for IMR-32 cells (Figure 3c). Following treatment, the ratio of cleaved to full-length PARP increased to a significantly greater extent in 2D-grown compared with 3D-grown cells (8-fold *versus* 2-fold and 10-fold *versus* 5-fold) for doxorubicin and vincristine, respectively (Figure 3d). In summary, our data suggest a decreased apoptotic response in 3D-grown tumor cells.

### Three-dimensional growth affects expression of autophagy-related genes in neuroblastoma cells

**Figure 4:**
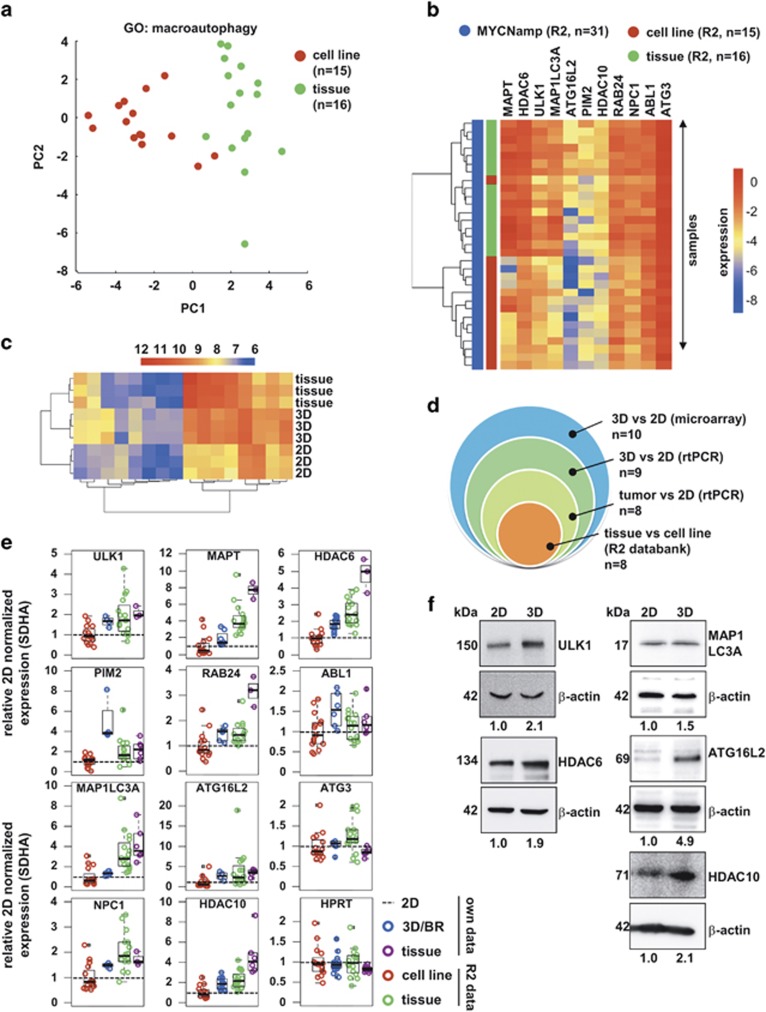
Elevated expression of autophagy-related genes in 3D cultures and tumors compared to 2D monolayer cultures. (**a**) Comparison of gene expression profiles from 15 *MYCN*-amplified neuroblastoma cell lines (red) and 16 *MYCN*-amplified neuroblastoma tissue samples (green) via the web-based R2 microarray database (http://r2.amc.nl) and the data set of mixed neuroblastoma cell lines/tissues (‘Versteeg’). The PCA was performed with all genes associated with the GO term ‘macroautophagy’. The first two principal components, PC1 and PC2, are shown. (**b**) Heatmap for validation of the TOP10 gene list with *MYCN*-amplified neuroblastoma cell lines (red) and *MYCN*-amplified neuroblastoma tissue samples (green) via the web-based R2 microarray database (http://r2.amc.nl) and the data set of mixed neuroblastoma cell lines/tissues (‘Versteeg’). (**c**) Heatmap showing expression of a subset of genes associated with the GO term 'autophagy' (GO:006914) in 2D- and 3D-cultured BE(2)-C cells and in neuroblastoma tumor tissue. Genes included are those with a component *P*-value less than 0.005 based on a globaltest analysis. Hierarchical clustering analysis, visualized as dendrograms on the left side of the heatmap, shows that the 2D samples cluster separately from the 3D and tumor samples. (**d**) Euler diagram displaying the overlap of significantly deregulated TOP10 genes in the three validation cohorts: 1) real-time PCR measurements of TOP10 genes in 3D- *versus* 2D- grown BE(2)-C cells; 2) real-time PCR measurements of TOP10 genes in three NB tumors (stage 4, *MYCN*-amplified) *versus* 2D- grown BE(2)-C cells; 3) web-based R2 microarray database (http://r2.amc.nl) with the data set of mixed neuroblastoma cell lines/tissues (‘Versteeg’). (**e**) Comparison of the expression of the TOP10 gene list plus *HDAC10* in BE(2)-C cells grown for 6 d in 2D (dotted normalization line; PCR) and 3D/3D-BR (blue; PCR) conditions, three neuroblastoma patient samples (tissue; magenta; PCR), as well as web-based (R2) microarray data from *MYCN* amplified neuroblastoma cell lines (red) and *MYCN*-amplified neuroblastoma tissue samples (green). All data were normalized to the respective 2D samples and neuroblastoma housekeeping gene *SDHA*. The PCR data were normalized in each experiment to 2D, thus the 2D expression values are equal to one across experiments. A second housekeeping gene, *HPRT*, served as normalization control. (**f**) Western blot presenting expression levels of the proteins ULK1, HDAC6, MAP1LC3A, ATG16L2, and HDAC10 in 2D- and 3D-cultured BE(2)-C cells. Numbers indicate expression relative to 2D and normalized to *β*-actin expression

Growth of tumor cells under spheroidal 3D conditions has been described to affect drug resistance via cellular pathways including proliferation, hypoxia and autophagy.^31, 32, 33^ As we have previously shown the importance of the autophagic degradation pathway in neuroblastoma cell cultures, we focused on genes associated with the Gene Ontology (GO) term ‘autophagy’ (GO:006914) and used the globaltest R package^34^ to check for differential regulation between 2D and 3D cultures. Differentially regulated genes were ranked according to the component *P*-value (Supplementary Table 3), and the ten genes with the lowest *P*-values (TOP10) were verified using semi-quantitative real-time PCR (Table 1). We additionally included *HDAC10* in the validation assays as this HDAC is also involved in the regulation of autophagic flux in neuroblastoma cells^12^ and is closely related to the TOP10 hit *HDAC6*. Nine of the TOP10 genes showed differences on RNA level concordant with the results of the gene expression analysis. Additionally, we re-evaluated gene expression data from the *MYCN* amplified neuroblastoma cell lines and tumors contained in the ‘Mixed Neuroblastoma’ data set (Versteeg; R2 database), performing a PCA using all genes associated with the GO term ‘macroautophagy’. Tissue samples and cell lines were clearly separated by the PCA (Figure 4a, Supplementary Figure S3a). Using the same data set, we performed a clustering analysis with the hit list of genes obtained from our own microarray analysis. It revealed an almost complete separation of cell lines from tumor samples (Figure 4b). A clustering analysis of our own microarray data using autophagy genes with a *P*-value less than or equal to 0.005 revealed two main clusters: 2D *versus* 3D/tumor tissue (Figure 4c). Of the nine PCR-validated genes, eight were significantly upregulated in tumor samples compared to 2D-cultured cell lines not only in our own microarray data, but also in the publically available R2 data set (Table 1,Figure 4d). A direct comparison of the TOP10 (plus *HDAC10*) gene expression among 2D- and 3D-grown cells with tumor tissue samples using our SDHA-normalized real-time PCR data as well as the SDHA-normalized Versteeg microarray data revealed that the gene expression of 3D-grown cells approaches that of tissue material (Figure 4e). To determine whether protein levels reflect our RNA expression profiles, we performed western blot analyses of ULK1, HDAC6, MAP1LC3A, ATG16L2 and HDAC10. Additional validation experiments were performed for *HDAC10,* as this candidate was not initially identified within the TOP10 list (Supplementary Figures S3b and c). Consistent with the upregulation of their transcripts, greater quantities of all five proteins were found in treatment-resistant 3D- compared to 2D-cultured BE(2)-C cells (Figure 4f).

### Three-dimensional cell culture increases autophagic flux

**Figure 5:**
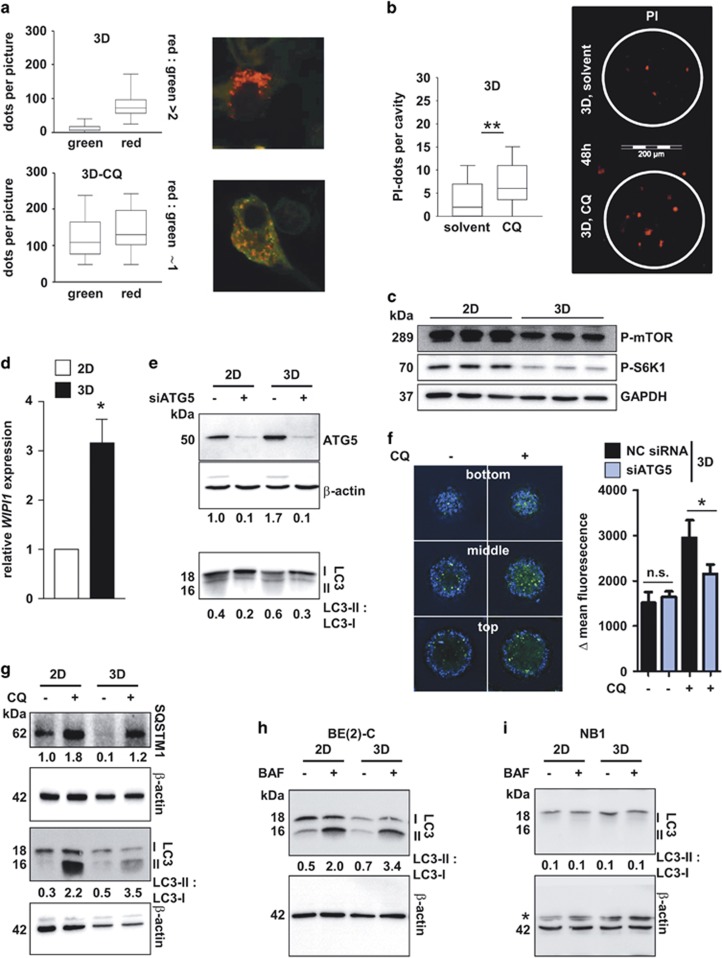
Detection of autophagic flux in cells grown under 3D culture conditions. (**a**) Fluorescence microscopic detection of autophagosome-lysosome fusion in stable mCherry-EGFP-LC3B BE(2)-C cells cultured under 3D conditions for 72 h. Yellow dots in the merged picture indicate autophagosomes only, whereas red-only signals indicate successful fusion to autophagolysosomes. CQ: chloroquine (25 *μ*M, 6 h). Graphs display ImageJ-based quantification of red or green dots/picture, respectively. Red-to-green ratio for 3D: 24.8±10.9 (S.E.M.). Red-to-green ratio for 3D plus CQ: 0.9±0.1 (S.E.M.). (**b**) In-chip fluorescence microscopic detection of dead BE(2)-C cells cultured under 3D conditions for 72 h. Red signals indicate propidium iodide (PI) positive cells. CQ: chloroquine (48 h). Graphs display ImageJ-based quantification of red dots/cavity. (**c**) Western blot displaying p-mTOR and P-S6K1 protein levels. BE(2)-C cells were grown as monolayers (2D) or in 3D culture for 7 days. GAPDH served as a loading control. (**d**) BE(2)-C cells were grown under 2D or 3D conditions and the mRNA expression levels of *WIPI1* were detected using real-time PCR as an indicator of autophagosome formation. *SDHA* and *HPRT* served as housekeeping genes. Bars represent mean values, error bars represent S.E.M. A one-sample *t*-test was performed to check whether the mean of 3D samples differs significantly from the value 1.0. *P<0.05. (**e**) Western blot displaying the conversion of LC3-I to LC3-II upon knockdown of ATG5 expression. Negative control transfected cells are indicated with a minus sign. BE(2)-C cells were grown as monolayers (2D) or in 3D culture. Numbers indicate ATG5 expression relative to negative control transfected 2D cells and normalized to *β*-actin expression (upper row) and the ratio of LC3-II to LC3-I (lower row). (**f**) Left: Representative pictures of CYTO-ID (green) and Hoechst (blue) co-stained 3D structures, treated with chloroquine (CQ), where indicated. Confocal microcopy pictures from the bottom, middle and top region of the 3D structure are presented. Right: Bar diagram. Quantification of CYTO-ID high cells (blank-corrected mean fluorescence) determined by FACS analysis. BE(2)-C cells were transfected with negative control (NC) or *ATG5* siRNA, respectively and treated with chloroquine (CQ), where indicated. (**g**) Western blot displaying SQSTM1 protein levels as well as the conversion of cytoplasmic LC3-I to autophagosome-associated LC3-II. Where indicated chloroquine (CQ, 25 *μ*M, 5 h) was added. BE(2)-C cells were grown as monolayers (2D) or in 3D culture. Numbers indicate SQSTM1 expression, relative to 2D and normalized to *β*-actin expression as well as the ratio of LC3-II to LC3-I. (**h**,**i**) Western blot displaying the conversion of LC3-I to LC3-II. Where indicated, bafilomycin A1 (BAF, 100 nM, 4 h) was added. BE(2)-C (**h**) and NB1 (**i**) cells were grown as monolayers (2D) or in 3D culture. Numbers indicate the ratio of LC3-II to LC3-I. *nonspecific band

It has been proposed that tumor cells adopt autophagy as a survival mechanism to cope with metabolic and cytotoxic stress.^15, 35, 36^ Similarly, spheroid cultures might use this survival strategy, especially in the inner starved zones.^33^ We examined autophagic flux in our standardized 3D-chip culture using stably transfected BE(2)-C cells expressing the tandem construct mCherry-EGFP-LC3B. Appearance of red dots indicates activated autophagic flux, characterized by successful fusion of autophagosomes with lysosomes, forming low-pH autolysosomes where acid-sensitive green fluorescence is lost. Three-dimensionally grown BE(2)-C cells clearly showed characteristic LC3 punctate staining with red mCherry, but not green EGFP fluorescence (Figure 5a). In contrast, EGFP fluorescence was detected upon treating 3D-grown cells with the flux-inhibiting lysosomotropic agent chloroquine (CQ), resulting in yellow punctae in the merged image (Figure 5a). Inhibition of autophagic flux by CQ also increased the amount of propidium iodide-positive dead cells inside the 3D structure (Figure 5b), further hinting at a pro-survival role of autophagy in spheroids. As an indicator for metabolic stress-induced autophagy and autophagosome formation, we analyzed mTOR activity and determined *WIPI1* mRNA expression levels^37^ as well as LC3-phosphatidylethanolamine (LC3-II) conjugation. Three-dimensionally grown BE(2)-C cells displayed decreased levels of p-mTOR, reflected in decreased phosphorylation of its substrate, S6K (Figure 5c, Supplementary Figure S3d), upregulated *WIPI1* (Figure 5d) and displayed an increased LC3-II/LC3-I-ratio compared to 2D cultures (Supplementary Figure S3e and f). Depletion of ATG5 inhibited autophagy initiation, detected by lack of LC3-II (Figure 5e, Supplementary Figure S3e). Moreover, BE(2)-C cell spheroids displayed CYTO-ID-positive cells^38^ in the inner region, which were further enriched upon treatment with CQ. Depletion of ATG5 decreased CYTO-ID labeling of autophagic compartments upon CQ treatment (Figure 5f). Inhibition of late autophagic flux through application of CQ (Figure 5g) or bafilomycin A1 (Figure 5h and Supplementary Figure S3f) increased the amount of LC3-II, further pointing towards an enrichment of autophagy under 3D conditions. In contrast, in drug-sensitive NB-1 cells (Figure 2f), LC3-II was undetectable and could not be induced by bafilomycin or 3D growth (Figure 5i), indicating a relative deficiency in autophagy initiation. In contrast to autophagy-competent BE(2)-C cells, ULK1, LC3A, ATG16L2 and HDAC6/10 were not enriched in 3D-grown NB-1 cells (Supplementary Figure S3g). Finally, 3D-grown BE(2)-C cells exhibited depletion of the autophagy substrate sequestosome 1 (p62/SQSTM1), which, together with SQSTM1 enrichment upon autophagy inhibition with CQ, implies more active autophagosomes (Figure 5g). These results indicate that 3D culture conditions enhance preexisting autophagic flux in autophagy-competent neuroblastoma cells.

### Transcriptional regulation of autophagy

**Figure 6:**
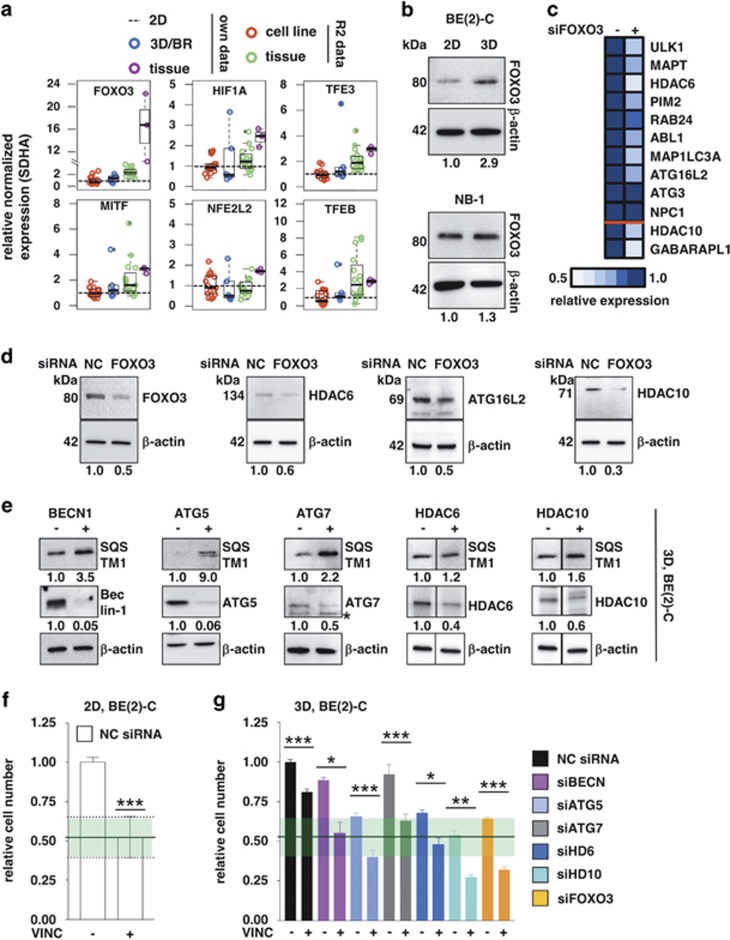
Transcriptional regulation of autophagy-related genes. (**a**) Boxplots comparing the expression of autophagy-related transcription factors in BE(2)-C cells grown for 6 days in 2D (dotted normalization line; Illumina) and 3D/3D-BR (blue; Illumina) conditions, three neuroblastoma patient samples (tissue; magenta; Illumina), as well as web-based (R2) microarray data from *MYCN*-amplified neuroblastoma cell lines (red) and *MYCN*-amplified neuroblastoma tissue samples (green). All data sets (web-based R2 as well as Illumina microarray expression data) were normalized to the respective 2D samples and neuroblastoma housekeeping gene *SDHA*. (**b**) Western blot presenting expression levels of FOXO3 in 2D- and 3D-cultured BE(2)-C cells or NB-1 cells, respectively. Numbers indicate expression relative to 2D and normalized to *β*-actin. (**c**) Heatmap displaying expression levels of TOP10 genes plus *HDAC10* and the well-known FOXO3 target gene *GABARAPL1* upon knockdown of FOXO3 expression in 3D-grown BE(2)-C cells 6 d after transfection and measured by real-time PCR. Negative control transfected cells are indicated with a minus sign. (**d**) Western blot: detection of FOXO3, HDAC6, ATG16L2 and HDAC10 protein levels in FOXO3 siRNA transfected 3D-grown BE(2)-C cells. *β*-Actin served as a loading control. Numbers indicate FOXO3, HDAC6, HDAC10 or ATG16L2 expression, respectively, relative to NC transfected cells and normalized to *β*-actin expression. NC: transfected with negative control siRNA. (**e**) Western blot displaying SQSTM1 protein levels of BE(2)-C cells grown as 3D cultures for 6 d upon knockdown of Beclin-1 (BECN), ATG5, ATG7, HDAC6 or HDAC10 expression. Negative control transfected cells are indicated with a minus sign. Numbers indicate SQSTM1 expression (upper row) or target expression (lower row) relative to negative control transfected 3D cells and normalized to *β*-actin expression. *nonspecific band (**f**) BE(2)-C cells were grown under 2D conditions for 6 d and treated with vincristine (10 ng/ml) where indicated for the last 96 h. (**g**) BE(2)-C cells were transiently transfected with siRNAs targeting Beclin-1 (siBECN, pink), ATG5 (light blue), ATG7 (grey), HDAC6 (siHD6, blue), HDAC10 (siHD10, mint green) or FOXO3 (orange), respectively. After transfection, cells were grown under 3D conditions for 6 d and treated with vincristine (10 ng/ml) where indicated for the last 96 h. (**f**,**g**) Relative cell number, meaning viable cells/ml normalized to solvent treated NC siRNA transfected control were determined with an automated cell counter. Green shading indicates effect ±S.E.M. of vincristine in 2D, NC siRNA transfected cells NC siRNA: transfected with negative control siRNA. Bars represent mean values, error bars represent S.E.M. Significant differences between groups were tested using an unpaired, two-tailed *t*-test. *P<0.05; **P<0.01; ***P<0.001

As elevated expression of autophagy genes points towards transcriptional upregulation, we investigated six transcription factors known to regulate the expression of autophagy-related genes.^39, 40, 41, 42^ All transcription factors, except NRF2 (NFE2L2), showed elevated expression in tumors compared to 2D cell lines. Our 3D cultures expressed four of the transcription factors at a level between that of 2D and tumor samples, showing that 3D growth alters expression in the same direction seen in tumors (Figure 6a). Additionally, *FOXO3* expression significantly correlated with the expression of seven TOP10 genes plus *HDAC10* (Supplementary Table 4). Three-dimensionally cultured BE(2)-C, but not NB-1 cells, displayed increased protein levels of FOXO3 (Figure 6b). Depletion of FOXO3 in 3D-cultured BE(2)-C cells altered expression of seven genes plus *HDAC10* as well as *GABARAPL1*, a well-known FOXO3 target gene^43^ (Figure 6c; exemplarily shown on protein level for HDAC6, ATG16L2 and HDAC10; Figure 6d).

We employed an siRNA-mediated knock-down approach to further validate the role of autophagy regulators and HDACs in mediating therapeutic resistance of 3D cultures. Three-dimensionally grown BE(2)-C cells depleted of *BECN1, ATG5, ATG7, HDAC6, HDAC10* and *FOXO3* exhibited impaired autophagic flux, demonstrated by SQSTM1 accumulation (Figure 6e) and increased chemotherapeutic sensitivity close to the level seen in 2D cultures, potentiating the effects of vincristine (Figures 6f and g), though *HDAC6* depletion achieved comparatively weak effects. The same approach also sensitized 2D-grown cells, however the combination exhibited no potentiation effect (except *HDAC10*; Supplementary Figure S4a). As 2D cell viability was not decreased by siRNA knockdown alone (except *HDAC10*), the more pronounced effects on viable cell number 6 d after transfection can be explained by the higher proliferation rate of 2D-grown cells (Supplementary Tables 2 and 5). Thus, transcriptional activation of autophagy supports enhanced expression of autophagy-related genes in 3D models and cancer tissues. Furthermore, the data suggest that interference with autophagy is a promising approach to break therapy resistance in neuroblastoma cells.

### Pharmacological inhibition of autophagic flux sensitizes 3D-grown neuroblastoma cells to cytotoxic treatment

Based on our findings that 3D-grown cells are sensitized to cytotoxic treatment when genetic interference disrupts autophagic flux, we explored whether a pharmacological block would yield similar results. To pharmacologically interfere with late-stage autophagy, we used chloroquine (CQ) and bufexamac, a class IIb HDAC inhibitor (Supplementary Figure S4b). Bufexamac impaired autophagic flux, demonstrated by an increased quantity of yellow mCherry-EGFP-LC3B dots, elevated SQSTM1 and LAMP-2 levels (Supplementary Figures S4c and d).

**Figure 7:**
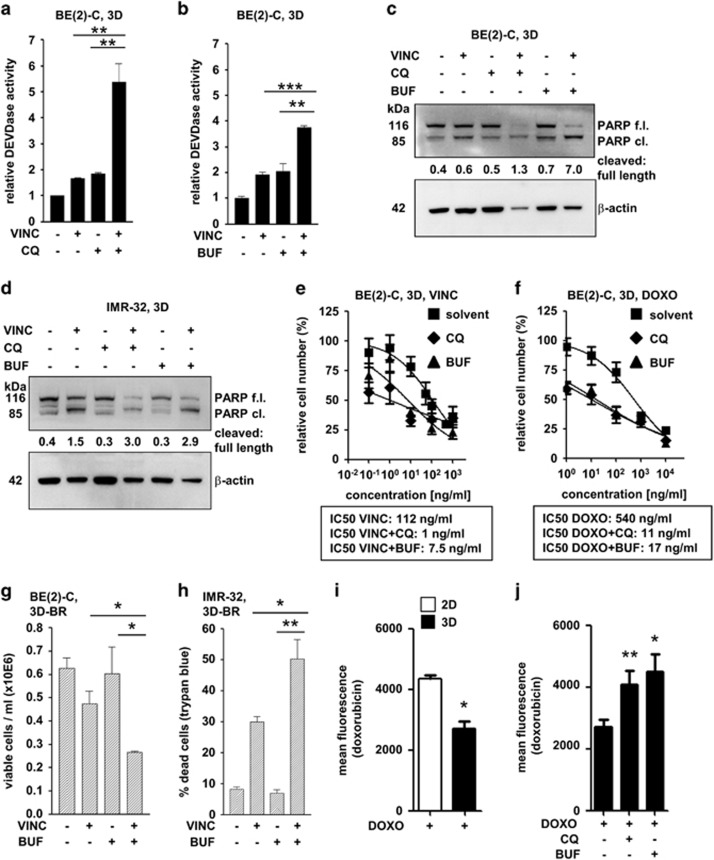
Pharmacological inhibition of autophagic flux sensitizes three-dimensional cell culture to treatment-induced cell death. (**a**) Cells were treated with chloroquine (25 *μ*M) and vincristine (10 ng/ml) for the last 48 h, where indicated. (**b**) Cells were treated with bufexamac (30 *μ*M) and vincristine (10 ng/ml) for the last 48 h, where indicated. (**a** and **b**) BE(2)-C cells grown under 3D-static conditions for 4 days. Caspase-3 (DEVDase) activity was measured using a fluorometric assay with activity (slope/min) measured relative to untreated cells. Bars represent mean values of at least three independent experiments, error bars represent S.E.M. Significant differences between groups were tested using an unpaired, two-tailed *t*-test. **P<0.01; ***P<0.001. (**c**,**d**) Western blot analysis of PARP cleavage in 3D-grown cells. Cells were cultured for a total of four days, with treatment starting 72 h (CQ, BUF) or 48 h (VINC) before lysate preparation. *β*-Actin served as a loading control. Numbers indicate the ratio of cleaved to full-length PARP. cl., cleaved; f.l., full-length. (**c**) BE(2)-C cells treated with vincristine (VINC, 10 ng/ml), chloroquine (CQ, 25 *μ*M) and bufexamac (BUF, 30 *μ*M) where indicated. (**d**) IMR-32 cells treated with vincristine (VINC, 3 ng/ml), chloroquine (CQ, 25 *μ*M) and bufexamac (BUF, 30 *μ*M) where indicated. (**e**-**f**) Three-dimensionally grown (3D) BE(2)-C cells were treated with chloroquine (CQ, 25 *μ*M, 72 h) or bufexamac (BUF, 30 *μ*M, 72 h) and vincristine (VINC, 48 h) (**e**) or doxorubicin (DOXO, 48 h) (f) in various concentrations. IC50 values were calculated with GraphPad Prism (function: log(inhibitor) *versus* normalized response; variable slope). Number of viable cells was assessed by automated cell counting and trypan blue exclusion and normalized to untreated cells. (**g**,**h**) One day after seeding, chips containing 3D-cultured BE(2)-C (**g**) or IMR-32 (**h**) cells were transferred into the bioreactor system (3D-BR) and medium or medium containing compounds was pumped through the system for 48 h. Where indicated, vincristine (VINC, 10 ng/ml, 48 h) or bufexamac (BUF, 30 *μ*M, 48 h) was applied. Results are displayed as means of at least three independent experiments. Error bars represent S.E.M. Significant differences between groups were tested using an unpaired, two-tailed *t*-test. *P<0.05; **P<0.01. (**i**) BE(2)-C cells were grown as 2D or 3D culture and treated with doxorubicin (0.5 *μ*g/ml) for 48 h. (**j**) BE(2)-C cells were grown as 3D culture, treated with doxorubicin (0.5 *μ*g/ml) for 48 h and co-treated with chloroquine (CQ, 5 *μ*M) or bufexamac (BUF, 30 *μ*M) where indicated. (**i**,**j**) Mean fluorescence was detected by flow cytometry. Bars represent mean values, error bars represent S.E.M. Significant differences between groups were tested using a paired, two-tailed *t*-test. **P<0.01; *P<0.05

Co-treatment of 3D-grown BE(2)-C cells with vincristine plus either CQ (Figure 7a) or bufexamac (Figure 7b) induced cell death, detected via effector caspase activity assay. Caspase-3 activation was further confirmed by detection of PARP cleavage in BE(2)-C (Figure 7c) and IMR-32 (Figure 7d) cells. Similarly, co-treatment of 3D-grown BE(2)-C cells with doxorubicin plus either CQ or bafilomycin further decreased viable cell number, with a greater potentiation effect in 3D-grown compared to 2D-grown cells (Supplementary Figures S4e–g).

The IC50 values for vincristine (100 ng/ml) and doxorubicin (540 ng/ml) were decreased 15- to 100-fold upon combination with CQ or bufexamac (Figures 7e and f). These results demonstrate that co-treatment is able to shift effective concentrations into a range that is clinically achievable in patient plasma (doxorubicin: approximately 60 ng/ml; vincristine: approximately 7 ng/ml).^44, 45^ Co-treatment of 3D cells transferred into the bioreactor system with bufexamac also significantly decreased the quantity of viable cells (Figure 7g) and increased cell death (Figure 7h).

Decreased intracellular doxorubicin, due to the 3D culture itself, the thereby increased autophagic flux, or a combination of both factors, could be one possible mechanism involved in resistance to cytotoxic treatment. Hence, we exploited the inherent fluorescence of doxorubicin to quantify intracellular doxorubicin by FACS in 2D and 3D cultures. Doxorubicin fluorescence was significantly decreased in 3D cultures (Figure 7i), while co-treatment with CQ and bufexamac reversed this effect (Figure 7j), yielding comparable levels achievable in 2D cultures (Figure 7i). Likewise, intracellular doxorubicin concentration was enriched when cells were depleted of FOXO3, ATG5 or HDAC10 (but not HDAC6) (Supplementary Figure S4h). These results demonstrate the substantial impact of autophagy on drug response at two levels: i. cell survival and ii. decreased intracellular drug concentration (as shown for doxorubicin), suggesting that pharmacological inhibition of autophagic flux effectively sensitizes 3D-grown cells to cytotoxic treatment.

## Discussion

Drug screens in 2D systems have successfully identified compounds that were subsequently translated into clinical use. Notable examples are inhibitors targeting tyrosine kinases, such as ALK (crizotinib), Bcr-Abl (imatinib) and HER2 (trastuzumab), which are aberrantly active due to genetic alterations.^46, 47, 48, 49^ Nevertheless, there is a poor correlation between effectiveness of emerging anti-cancer agents in cell culture and ultimate treatment success rates in patients, which are low and need improvement.^50^ Culture conditions impact the regulation of cell fate and responsiveness to external stimuli. Two-dimensional cultures are favored due to high-throughput testing capability, but may lose phenotypic and functional characteristics,^5^ which can be overcome by culturing cells under more physiological 3D conditions. A remaining practical challenge is large-scale testing of compound libraries, under reproducible 3D conditions. The chip-based system used here yields 3D cultures with a well-defined and consistent geometry, stable and comparable culture conditions, and compatibility with a bioreactor system, allowing for an even more representative environment.

Several DNA microarray studies demonstrated significant, tumor-relevant molecular changes induced by 3D growth.^3^ Our gene expression studies corroborate these results and show that 3D growth affects the expression of ATG genes and autophagy-controlling transcription factors, such as TFEB and FOXO3. FOXO transcription factors are regulated by a wide range of external stimuli, including nutrients and oxidative stress,^51^ and can support stress resistance through induction of antioxidant as well as ATG proteins.^52^ Our results indicate that autophagy and the expression of ATG genes may be induced as an adaptive survival strategy due to stress conditions inside the 3D structure, which is also reflected in strongly reduced mTOR activity. Several publications indicate that there is cross-talk between the activity of the mTOR complexes and the activity of FOXO3a via AKT, which can be activated by mTORC2.^53^ AKT, in turn, negatively regulates FOXO3a by phosphorylation, preventing its nuclear translocation.^54^ Thus, it is conceivable that reduced activity of the mTORC/AKT axis supports FOXO3 transcriptional activity in our model system. Overall, autophagy induction protects the cells, as co-treatment with autophagic flux blockers increases sensitivity of 3D cultures to chemotherapy. This is in line with several studies demonstrating that inhibition of autophagy sensitizes a diversity of tumor entities towards chemotherapeutic drugs *in vitro* and *in vivo*.^55, 56, 57, 58^ Accordingly, several early phase clinical trials are currently under way to evaluate autophagy inhibition using (hydroxy-)chloroquine in combination with chemotherapy or targeted agents.^58, 59^ Unraveling the exact mechanism as to how autophagy is protecting which type of tumor cell is the subject of current research, which also indicates a particular role for lysosomes as mediators of drug resistance.^60^

Although technically different 3D culture systems are similar in terms of more tissue-like cell morphology,^3^ the role of autophagy might depend on tumor entity, tumor stage and 3D culture context. For example, in contrast to our 3D model, the 3D laminin-rich ECM model resulted in increased sensitivity to doxorubicin and compromised autophagy.^61^

Accurate determination of susceptibility to treatment is crucial for preclinical assessment of a compound’s efficacy. In our setting, 2D-grown BE(2)-C cells are sensitive to doxorubicin and vincristine in clinically achievable concentrations, while the 3D model predicts resistance.^44, 45^ Consistent with the latter, the BE(2)-C cell line was isolated from a relapsed neuroblastoma patient following multi-agent chemotherapy, including doxorubicin.^62^ Cytotoxic drugs combined with autophagy inhibitors shifted the effective chemotherapy concentrations back to clinically achievable levels. We observed, at least in the case of doxorubicin, that sensitization is accompanied by an increased uptake (or decreased efflux) of the cytotoxic drug into the 3D structure and that inhibition of autophagy plays an important role in this effect. However, we cannot exclude that additional, potentially drug-specific mechanisms account for the beneficial effects of combination treatment. Overall, 3D models decrease the discrepancy between cell culture and tumor tissue and are useful for the identification of points of vulnerability for cancer treatment. Our work with 3D cultures underscores two critical aspects, which are (at least partly) linked together: (i) the upregulation of cell-protective autophagy and (ii) decreased intracellular drug concentration.

Altogether, our data favor 3D cell cultures as tissue-like models, recapitulating solid tumors with respect to cell morphology, gene expression and cellular functions. The inclusion of 3D models is highly advantageous, particularly for mechanistic studies and investigations of drugs targeting the autophagic pathway.

## Materials and methods

### 3D and 2D cell cultures and patient samples

Human neuroblastoma cell lines BE(2)-C (ECACC), IMR-32 (DSMZ) and the tetracycline-inducible *shMYCN* IMR5/75^63^ (generously provided by the laboratory of F. Westermann) were cultured under standard conditions (DMEM with L-glutamine and 4.5 g/l glucose containing 10% FCS (Sigma) and 1% non-essential amino acids (NEAA; Invitrogen, Darmstadt, Germany)). Human neuroblastoma cell lines SK-N-BE, NB-1 (#RCB1953, RIKEN cell bank, Japan) and Kelly (DSMZ) were cultured in RPMI with l-glutamine containing 10% FCS (Sigma, Taufkirchen, Germany) and 1% non-essential amino acids (NEAA; Invitrogen). Wildtype and HDAC10-knockout HAP1 cells (Horizon Discovery Group, Cambridge, UK) were cultured in Iscove's Modified Dulbecco's Medium (IMDM) with 10% FCS (Sigma). All cell lines were regularly checked for contamination (Multiplexion, Heidelberg, Germany) and verified using DNA fingerprinting authentication by the DSMZ, Germany. The 3D cultures were prepared on ridged scaffolds (MatriGrid structures), with a total active area of 5 × 5 mm^2^, as described previously.^9, 64^ Briefly, the microcavity-containing polymeric scaffolds, each with a diameter of 300 *μ*m and a depth of 207 *μ*m,^64^ were coated with collagen type I (10 *μ*g/cm^2^, Sigma, #C3867) as substratum, allowing cells to adhere to the scaffold. After 2 h of drying, cell solution was added to the prepared chip. For the static 3D model, cells were cultured on 3D scaffolds for 3–7 days, as indicated in the respective figure legend. For the bioreactor system, cells were cultured on 3D scaffolds for 2 days, then the cell-containing chips were inserted into the bioreactor cube^9, 64^ and cultured for another 2–5 days, as indicated in the respective figure legend. Complete medium containing test compound or solvent control was pumped through the bioreactor system (Ismatec, Wertheim, Germany) with a flow rate of 25–35 *μ*l/min. If necessary, the spheroids were recovered from the cavities by trypsinization (3–4 min at 37 °C) and one to maximum two very careful washing steps with fresh medium.

#### Generation of stable cells

Human BE(2)-C cell lines stably expressing mCherry-EGFP-LC3B were established by transfection using Effectene (Qiagen, Hilden, Germany) with the mCherry-EGFP-LC3B construct (no. 22418; Jayanta Debnath) from Addgene. Transfected cells were selected with puromycin (2 *μ*g/ml) for 3 weeks. A mixed population of puromycin-resistant cells was used for experimental analysis.

#### Patient samples

The three neuroblastoma patient samples from the German Neuroblastoma Trials NB90-NB2004^30^ were obtained from the German Society of Pediatric Oncology and Hematology Tumor Bank and Neuroblastoma Study Center (Cologne, Germany). All of the patients were registered with the German neuroblastoma study and written informed consent was obtained.^30^ The three tumor samples were chosen to resemble the characteristics of BE(2)-C cells, which are derived from a stage 4, *MYCN* amplified, 1p-deleted, undifferentiated tumor of a 2-year-old child. Tumor sample #1: stage 4, *MYCN* amplified, 1p-deleted, undifferentiated, age at diagnosis: 279 days. Sample #2: stage 4, *MYCN* amplified, 1p-deleted, poorly differentiated, age at diagnosis: 121 days. Sample #3: stage 4, *MYCN* amplified, 1p-deleted, undifferentiated, age at diagnosis: 536 days.

### Reagents and transfection

Bufexamac (100 mM stock),^65^ bafilomycin A1 (10 *μ*M stock) and rapamycin (1 mM stock) were obtained from Sigma and dissolved in DMSO. Chloroquine (Sigma; 100 mM stock), doxorubicin (Biozol, Eching, Germany; 1 mg/ml stock), and vincristine (Enzo Life Sciences, Lörrach, Germany, 5 mM stock) were dissolved in H_2_O. Cisplatin (Axxora, Lörrach, Germany; 15 mg/ml stock), etoposide (Enzo Life Sciences; 10 mg/ml) and crizotinib (Selleckchem, Houston, USA; 10 mM), were dissolved in DMSO. Commercially available siRNAs were used for transient transfections (pooled): HDAC10 #33581 and #120681 (Ambion, Huntingdon, UK), HDAC6 #120451 and #120450 (Ambion), ATG5 HS_APG5L_6 and HS_APG5L_3 FlexiTube siRNA (Qiagen, Hilden, Germany), ATG7 Hs_APG7L_5 FlexiTube siRNA (Qiagen), FOXO3 #s5260, s5262, s5261 (pooled, Ambion) and BECN1: Hs_BECN1_5 FlexiTube siRNA (Qiagen). The following sequence was additionally chosen to specifically target BECN1: 5′-CAG UUU GGC ACA AUC AAU Att-3′ (Beclin 1 sense); 5′-UAU UGA UUG UGC CAA ACU Gtt-3′ (Beclin 1 antisense). The NC siRNAs (Silencer Negative Control #1 and Silencer Negative Control #5; Ambion) were used as negative controls. Transfection was performed as described previously^12^ in 2D monolayer cultures and cells were transferred 24 h after transfection into the 3D model. The percentage of siRNA-transfected BE(2)-C cells was 90% as determined by fluorescently-labeled siRNA siGLO Lamin A/C (Dharmacon).

### Immunoblotting and fluorescent microscopy

Western blot analysis was performed as previously described.^66^ The following antibodies were used: anti-LC3B (L7543; Sigma), anti-p62/SQSTM1 (MBL-M162-3B), anti-HDAC6 (sc-11420, Santa Cruz), anti-HDAC10 (H3413, Sigma), anti-PARP (4C10-5; BD Pharmingen, Heidelberg, Germany), anti-Beclin-1 (D-18; Santa Cruz Biotechnology, Santa Cruz, CA, USA), anti-FOXO3A (2497 s, Cell Signaling), anti-LAMP2 (H4B4; Santa Cruz Biotechnology), anti-acetylated tubulin (6-11B-1; Sigma), anti-GAPDH (JC1682928, Millipore, Darmstadt, Germany), anti-ULK1 (#8054; Cell Signaling), anti-ATG16L2 (AP11695c-AB; Abgent), anti-MAP1LC3A (AP1805a; Abgent, San Diego, CA, USA), anti-ATG5 (#2630; Cell Signaling, Leiden, Netherlands), anti-ATG7 (#2631, Cell Signaling), anti-p-mTOR (Ser2448; Upstate), anti-p-S6K1 (Thr412; Upstate) and anti-β-actin (clone AC-15; Sigma). Ratios were calculated with the Bio-1D Version 12.10a software (Peqlab, Erlangen, Germany).

#### Fluorescence microscopy

Stable mCherry-EGFP-LC3B BE(2)-C cells were viewed using the × 63 objective on a Zeiss LSM700 laser scanning confocal microscope. For quantification, the Image-based Tool for Counting Nuclei (ITCN) in ImageJ software (U. S. National Institutes of Health, Bethesda, MD, USA; http://imagej.nih.gov/ij/) was used on 8-bit pictures (threshold adjusted to 15, 255 for green dots and 25, 255 for red dots).

#### Imaging of CYTO-ID stained spheroids

BE(2)-C cells were seeded in 3D scaffolds and grown for 2 days. On the night before treatment, chips were transferred to 8-well *μ*-Slides (Ibidi) and treated with control medium (DMEM w/o phenol-red 10%FCS 1% NEAA) or 5 *μ*M CQ. Slides were carefully washed twice with 1x Assay Buffer+5% FCS and stained with 200 *μ*l/well CYTO-ID staining solution (1 × assay buffer, 5% FCS, 2 *μ*l CYTO-ID, 1 *μ*l Hoechst 33352; Enzo Life Sciences) for 30 min at 37 °C. Chips were carefully washed and imaged on a Zeiss LSM710 confocal microscope (Jena, Germany).

#### Immunohistochemistry and nuclear staining

Immunohistochemistry and nuclear staining were performed on BE(2)-C cells grown under 2D or 3D conditions, and a neuroblastoma tissue sample. Briefly, cells were fixed with ice-cold methanol for 20 min and stained with hematoxylin and eosin (HE) or diamidino-2-phenylindole (DAPI). Before staining, spheroids were freed from the polymeric chip by trypsinization (4 min at 37 °C), followed by careful transfer onto glass slides after addition of medium. The neuroblastoma tissue sample (#14T1OL) was provided by the tissue bank of the National Center for Tumor Diseases (NCT, Heidelberg, Germany) in accordance with the regulations of the tissue bank and with the approval of the ethics committee of Heidelberg University. Sections were deparaffinized, incubated with Tris-buffer saline containing 0.5% Triton X-100 (TBST) for 20 min at room temperature. Following staining with HE or Hoechst dye (30 min at room temperature), the slides were washed and mounted. Fluorescent images were acquired on a Zeiss LSM 710 confocal microscope using the 40 × objective.

#### Quantification of CYTO-ID via flow cytometry

Three days after siRNA transfection, BE(2)-C cells were seeded in 3D scaffolds and grown for an additional 72 h. Where indicated, cells were treated with 5 *μ*M CQ the night before staining with CYTO-ID (Enzo Life Sciences). For staining (6 d post-siRNA transfection), chips were washed once with medium w/o phenol red, transferred to a fresh 6-well plate and covered with 500 *μ*l CYTO-ID staining solution (1 × assay buffer, 5% FCS, 1 *μ*l CYTO-ID) for 30 min at 37 °C. Cells were washed with medium w/o phenol red, trypsinized (4 min. 37 °C), centrifuged and washed again in cold medium w/o phenol-red. CYTO-ID fluorescence was quantified on a BD FACSCanto II platform using an Alexa488 filter setting

#### Quantification of doxorubicin uptake

BE(2)-C cells were seeded in 3D scaffolds 24 h prior to treatment. Spheroids were treated for 48 h with solvent control (DMSO), doxorubicin (500 ng/ml), combinations of doxorubicin with bufexamac (30 *μ*M), chloroquine (5 *μ*M) or both. For the knockdown approach, BE(2)-C cells were seeded in 3D scaffolds three days after transfection with ATG5, FOXO3a, HDAC6, HDAC10 or control siRNAs, grown for an additional 48 h and treated with 500 ng/ml doxorubicin 24 h before staining. For FACS analysis, chips were washed with RPMI w/o phenol red (10% FCS), transferred to a fresh 6-well plate, trypsinized (4 min, 37 °C), centrifuged and washed again in cold medium w/o phenol-red (10% FCS). For the analysis of 2D conditions, BE(2)-C cells were seeded in monolayers in six-well plates 24 h prior to treatment. Thereafter, the same protocol as for the 3D scaffolds was followed. Doxorubicin fluorescence was quantified on a BD FACSCanto II platform using a Phycoerythrin (PE) filter setting.

### Cell counting, cell viability and cell death assays

For cell death detection, adherent as well as floating cells were collected.

#### Viability assay

Trypsinized cells were pooled with corresponding supernatant, centrifuged and resuspended in 1 ml cell culture media. Cell viability was measured by automated trypan blue staining with the Vi-Cell XR Cell Viability Analyzer from Beckman Coulter (Krefeld, Germany).

#### Nicoletti staining

Cell death quantification by flow cytometry was performed as described previously.^67^

##### Caspase protease activity assay

DEVDase or IETDase activity was measured with the Caspase-3 or Caspase-8 Fluorometric Assay (BioVision), respectively, according to the manufacturer’s protocol.

##### PARP cleavage

For detection of Poly(ADP-ribose) polymerase (PARP) cleavage, anti-PARP (4C10-5; BD Pharmingen) was used.

### RNA-Isolation, microarray analysis and real-time PCR

Total RNA was isolated from three independent human neuroblastoma tissue samples (see above) or 2D, 3D-static and 3D bioreactor neuroblastoma cell cultures (each in triplicate), using the RNeasy MiniKit (Qiagen). For microarray analysis, 1 *μ*g RNA per sample was used. Gene expression analysis was performed at the house-internal Genomics and Proteomics Core Facility using human whole genome HT-12 v4 BeadChips. Normalization of the raw intensity data was performed by the microarray unit of the DKFZ Genomics and Proteomics Core Facility with Illumina BeadStudio Data Analysis Software version v4_r2. The normalized gene expression profiles were further analyzed by principal components analysis (PCA) using the statistical software R.^68^ For analysis of autophagy transcription factor expression the following probesets were applied: FOXO3: ILMN_1844692; HIF1A: ILMN_2379788; MITF: ILMN_2304186; MYCN: ILMN_1653761; NFE2L2: ILMN_1790909; TFE3: ILMN_1764826; TFEB: ILMN_1733616; SDHA: ILMN_1744210. Real-time RT-PCR was performed as described previously with at least three biological replicates and two technical replicates.^66^ Data were normalized against neuroblastoma housekeeping genes *SDHA* and *HPRT*^69^ and set in relation to negative control. The following specific primer pairs were used: *ABL1* (forward: 5′-TTGACCAAGCCTCTACAGGG-3′, reverse: 5′-AGACCCGGAGCTTTTCACCT-3′), *ATG3* (forward: 5′-GACCCCGGTCCTCAAGGA A-3′, reverse: 5′-TGTAGCCCATTGCCATGTTGG-3′), *ATG16L2* (forward: 5′-TGGACAAGTTCTCAAAGAAGCTG-3′, reverse: 5′-CCTAGTGCGACCAGTGAT-3′), *HDAC6* (forward: 5′-CAAGGAACACAGTTCACCTTCG-3′, reverse: 5′-GTTCCAAGGCACATTGATGGTA-3′), *HDAC10* (Primer #1: forward: 5′-CTCACTGGAGCTGTGCAAAA-3′, reverse: 5′-GATCCTGTGTAGCCCGTGTT-3′ Primer #2: forward: 5′-ATCTCTTTGAGGATGACCCCAG-3′, reverse: 5′-ACTGCGTCTGCATCTGACTCTC-3′ Primer #3: forward: 5′-CAGTTCGACGCCATCTACTTC-3′, reverse: 5′-CAAGCCCATTTTGCACAGCTC-3′), *HPRT* (hypoxanthine phosphoribosyltransferase 1, forward: 5′-TGACACTGGCAAAACAATGCA-3′ reverse: 5′-GGTCCTTTTCACCAGCAAGCT-3′), *MAP1LC3A* (forward: 5′-AACATGAGCGAGTTGGTCAAG-3′, reverse: 5′-GCTCGTAGATGTCCGCGAT-3′), *MAPT* (forward: 5′-GATTGGGTCCCTGGACAATA-3′, reverse: 5′-GTGGTCTGTCTTGGCTTTGG-3′), *NPC1* (forward: 5′-GCACCTTTTACCATCACTCCTG-3′, reverse: 5′-GGCCACAGACAATAGAGCAGT-3′), *PIM2* (forward: 5′-TTGACCAAGCCTCTACAGGG-3′, reverse: 5′-CCACCTGGAGTCGATCTGTGA-3′), *RAB24* (forward: 5′-TACGTGGGCAAGACTAGCCT-3′, reverse: 5′-GCCCCGATGGTGTTCTGATAAG-3′), *SDHA* (succinate dehydrogenase complex, subunit A, forward: 5′-TGGGAACAAGAGGGCATCTG-3′, reverse: 5′-CCACCACTGCATCAAATTCATG-3′) and *ULK1* (Qiagen: QT00009884).

### *In vitro* HSP70 deacetylation activity assay

The inhibitory activity of bufexamac towards HDAC-mediated HSP70-peptide deacetylation was measured according to the HDAC activity protocol.^66, 70^ Briefly, an equal amount of BE(2)-C cell lysate (6.5 *μ*g) per sample was used as an enzyme source. The substrate SQRQATK(Ac) (final concentration: 10 *μ*M) was synthesized and conjugated with AMC by Peps4LS (Heidelberg, Germany). The HDAC reaction was performed at 37 °C for 30 min before adding the developer reagent. The free AMC was detected after 30 min of incubation time with excitation of 380 nm and emission 460 nm at kinetic mode for 10 min.

### Web-based gene expression analysis

R2 (R2: microarray analysis and visualization platform; http://r2.amc.nl) was used for principal component analysis (PCA) and to investigate expression of the TOP10 regulated autophagy-related genes in a publically available mixed cohort of primary neuroblastoma patients (Academic Medical Center (AMC) - Versteeg; Gene Expression Omnibus (GEO) database accession no. GSE16476, chiptype u133p2) and neuroblastoma monolayer cell lines (Academic Medical Center (AMC) - Versteeg; Gene Expression Omnibus (GEO) database accession no. GSE28019, chiptype u133p2). The subset of tumors and cell lines with *MYCN* amplification was used and the following probesets were applied: *ABL1*: 202123_s_at; *ATG16L2*: 229389_at; *ATG3*: 221492_s_at; *FOXO3*: 224891_at; *HDAC6*: 206846_s_at; *HDAC10*: 226672_s_at; *HIF-1 A*: 200989_at; *HPRT*: 202854_at; *MAP1LC3A*: 227219_x_at; *MAPT*: 225379_at; *MITF*: 226066_at; *MYCN*: 209757_s_at; *NFE2L2*: 201146_at; *NPC1*: 202679_at; *PIM2*: 204269_at; *RAB24*: 225251_at; *SDHA*: 201093_x_at; *TFE3*: 212457_at; *TFEB*: 50221_at; *ULK1*: 209333_at. Patient characteristics were published previously.^71^ A representative picture for an HE-stained patient sample (#itcc0056) is provided in Supplementary Figure S1d, indicating a high tumor-to-stroma ratio (source: http://r2.amc.nl). Comparison of gene expression level between tissue and cell line samples from the R2 neuroblastoma mixed database was performed on data normalized to SDHA expression using a two-tailed unpaired *t*-test using the statistical software R (R version 3.2.1, 2015; The R Foundation for Statistical Computing). Gene expression was normalized to the neuroblastoma housekeeping gene, *SDHA*, to maintain comparability with our real-time PCR analysis (see above).

### Statistical analysis

Principal component analysis (PCA) was used to visually assess similarities and differences between samples and determine whether samples can be grouped. The analysis was performed by applying a singular value decomposition to the mean-centered gene expression values of the 1000 genes showing highest variability as determined by fitting a linear model using the limma package in R.^72, 73^

In order to identify pathways that are differentially regulated between 2D and 3D (static and bioreactor system) cell cultures, we applied a globaltest.^34^ This test evaluates Gene Ontology (GO) terms (http://www.geneontology.org/) of the ‘Biological Process’ ontology.

In addition, we sorted the regulated genes with the GO term ‘autophagy’ (GO:006914) by component *P*-value to identify the TOP10 genes, meaning the ten genes with lowest component *P*-value. These component *P*-values reflect the contribution of each gene to the global test statistic and serve here as an explorative tool to rank genes. They cannot be used to control a type one error rate or make inferential decisions and therefore we leave them unadjusted for multiplicity. These calculations were performed using the Bioconductor Version 2.10 released 2 April 2012.^74^

Heatmaps showing gene expression were generated using the package pheatmap in R (Raivo Kolde (2015). pheatmap: Pretty Heatmaps. R package version 1.0.7. http://CRAN.R-project.org/package=pheatmap). Dendrograms shown for the heatmaps reflect the result of a hierarchical clustering analysis, which was performed using Ward’s method, with an algorithm that preserves Ward’s criterion.

IC_50_/EC_50_ values were calculated with GraphPad Prism version 5.00 for Windows, GraphPad Software, San Diego California USA, www.graphpad.com. Each experiment was repeated at least thrice. For statistical analysis of cell culture experiments, a two-tailed *t*-test of significance was performed to compare treatments using GraphPad Prism. For real-time PCR analysis, all samples were normalized to 2D samples (=1.0) and a one-sample *t*-test was used to test whether means of 3D samples or tissue samples, respectively, are significantly different from a hypothetical value (1.0), using GraphPad Prism version 3.0a. *P*-values less than 0.05 were considered significant (***P<0.001; **0.001⩽*P*<0.01; *0.01⩽*P*<0.05).

## Publisher’s Note

Springer Nature remains neutral with regard to jurisdictional claims in published maps and institutional affiliations.

## Figures and Tables

**Table 1:** TOP10 of regulated autophagy genes.

**Gene name**	**3D**^a^ ***versus* 2D Illumina Array ‘autophagy’ (GO:006914) *P*-value**		**3D**^a^ ***versus* 2D real-time PCR (SDHA normalized) *P*-value**^b^		**Tumor**^c^ ***versus* 2D real-time PCR (SDHA normalized) *P*-value**^b^		**Tissue**^d^**versus cell line**^e^ **R2 database (SDHA normalized) *P*-value**		**Tissue**^d^ **versus cell line**^e^ **R2 database *P*-value**	
*ULK1*	<0.0001	↑	0.0108	↑	0.0206	↑	0.0009	↑	n.s.	—
*MAPT*	<0.0001	↑	0.0404	↑	0.0070	↑	<0.0001	↑	<0.0001	↑
*HDAC6*	0.0003	↑	0.0002	↑	0.0236	↑	<0.0001	↑	<0.0001	↑
*PIM2*	0.0003	↑	0.0379	↑	0.0321	↑	0.0085	↑	n.s.	—
*RAB24*	0.0005	↑	0.0219	↑	0.0239	↑	0.0021	↑	n.s.	—
*ABL1*	0.0006	↑	0.0268	↑	n.s.	—	n.s.	—	0.02	↓
*MAP1LC3A*	0.0006	↑	0.0175	↑	0.0067	↑	<0.0001	↑	<0.0001	↑
*ATG16L2*	0.0009	↑	0.0474	↑	0.0006	↑	0.0008	↑	0.0095	↑
*ATG3*	0.0010	↓	n.s.	—	0.0225	↓	n.s.	—	0.0013	↓
*NPC1*	0.0012	↑	0.0210	↑	0.0439	↑	<0.0001	↑	0.0057	↑
*HDAC10*	n.d.		0.0011	↑	0.0075	↑	<0.0001	↑	0.0008	↑

Abbreviations: n.d., not determined; n.s., not significant

a 3D comprises 3D and 3D bioreactor samples

b One-sample *t*-test to test whether means of 3D samples or tumor samples, respectively, are significantly different from 1.0

c Three individual neuroblastoma patient samples with characteristics resembling BE(2)-C cells (stage 4, MYCN amplified, 1p-deleted, undifferentiated)

d MYCNamp neuroblastoma tissue samples (*n*=16) from data set of mixed neuroblastoma cell lines/tissues (Versteeg) contained in the web-based R2 microarray database (http://r2.amc.nl)

e MYCNamp neuroblastoma cell lines (*n*=15) from data set of mixed neuroblastoma cell lines/tissues (Versteeg) contained in the web-based R2 microarray database (http://r2.amc.nl)
