# Supplementary material for: Three-dimensional tumor cell growth stimulates autophagic flux and recapitulates chemotherapy resistance
Source: Cell Death Dis. 2017 Aug 24;8(8):e3013–. doi: 10.1038/cddis.2017.398 (PMC5596581; doi:10.1038/cddis.2017.398)
Supplement: Supplementary Figure Legends [file cddis2017398x1.docx]

**SUPPLEMENTARY FIGURE LEGENDS**

Supplementary Figure 1:

**Characterization of the neuroblastoma three-dimensional cell growth model**

**(a)** Principal component analysis (PCA) of transcriptomes, using gene expression profiles from 15 *MYCN* amplified neuroblastoma cell lines (red) and 16 *MYCN* amplified neuroblastoma tissue samples (green) via the web-based R2 microarray database (http://r2.amc.nl) and the dataset of mixed neuroblastoma cell lines/tissues (‘Versteeg’). **(b)** Scheme of 3D-bioreactor system. BR: bioreactor. **(c)** Pictures of the bioreactor: the closed system is shown on the left, the open system displaying the scaffold holding chamber is shown on the right. BR: bioreactor. **(d)** HE staining of tissue sample #itcc0056 from the publically available cohort of primary neuroblastoma patients (Academic Medical Center (AMC) - Versteeg; Gene Expression Omnibus (GEO) database accession no. GSE16476; chiptype u133p2; http://r2.amc.nl). Upper panel, 10x magnification. Lower panel, 40x magnification. **(e)** HE staining of IMR-32 cells grown as monolayers under normal 2D conditions or in 3D culture (polymeric chips). Cells grown for 72 h under 3D conditions were freed from the chip for staining. **(f)** Principal component analysis (PCA) of transcriptomes from 2D (open), 3D-static (3D; black), 3D-bioreactor (3D-BR; blue) 6 d cultures of BE(2)-C cells and three neuroblastoma patient samples (tissue; magenta).

Supplementary Figure 2:

**Three-dimensional cell growth affects drug responsiveness of neuroblastoma cells**

IMR-32 cells grown in monolayer (2D) or in 3D were treated 24 h post-seeding with doxorubicin **(a)** or vincristine **(b)** in various concentrations for 48 h. EC50 values based on percentage of dead cells were calculated with GraphPad Prism.

Supplementary Figure 3:

**Enhancement of autophagy in 3D-cultures of autophagy competent cells**

**(a)** Comparison of gene expression profiles from 15 *MYCN* amplified neuroblastoma cell lines (red) and 16 *MYCN* amplified neuroblastoma tissue samples (green) via the web-based R2 microarray database (http://r2.amc.nl) and the dataset of mixed neuroblastoma cell lines/tissues (‘Versteeg’). The PCA was performed with all genes associated with the GO term ‘macroautophagy’. **(b)** Detection of *HDAC10* expression in 2D- and 3D-grown cells as well as tumor tissue samples by realtime PCR with three different sets of primer pairs. **(c)** Western Blot for the detection of HDAC10 in cell lysates of HAP1 wild-type (indicated by a minus) and HAP1 *HDAC10*-knockout cells (indicated by a plus sign). Actin served as a loading control. **(d)** Western blot displaying P-mTOR and P-S6K1 protein levels. BE(2)-C cells were grown as monolayers (2D) or in 3D culture for 4 days and treated with rapamycin (RAPA, 100 nM) for the last 2 h, where indicated. Actin served as a loading control. **(e)** Quantification of three biological replicates of the conversion of LC3-I to LC3-II upon knockdown of ATG5 expression (siATG5). Negative control transfected cells are indicated with a minus sign. BE(2)-C cells were grown as monolayers (2D) or in 3D culture. **(f)** Quantification of three biological replicates of the conversion of LC3-I to LC3-II. Where indicated, bafilomycin A1 (BAF, 100 nM, 4 h) was added. BE(2)-C cells were grown as monolayers (2D) or in 3D culture. **(g)** Western blot showing expression levels of the proteins ULK1, HDAC6, MAP1LC3A, ATG16L2, and HDAC10 in 2D- and 3D-cultured NB-1 cells. Numbers indicate expression relative to 2D and normalized to β-actin expression.

Supplementary Figure 4:

**Genetic and pharmacological inhibition of autophagic flux sensitizes autopohagy competent cells to treatment-induced cell death**

**(a)** Left panel: BE(2)-C cells were transiently transfected with siRNAs targeting Beclin-1 (siBECN, pink), ATG5 (light blue), ATG7 (grey), HDAC6 (siHD6, blue), HDAC10 (siHD10, mint green) or FOXO3 (orange), respectively. Cells were grown under classical 2D conditions for 6 d and treated with vincristine (10 ng/ml), where indicated, for the last 96 h. NC siRNA: negative control siRNA. Right panel: Assessment of the relative potentiation effect induced via siRNA treatment. Sensitization to vincristine was calculated as x-fold potentiation through dividing the normalized decrease in viable cell number for each target by the decrease of viable cell number obtained by vincristine treatment of control siRNA transfected cells. **(b)** Western blot depicting acetyl-tubulin levels. Where indicated, bufexamac (BUF, 30 µM) was added for the last 24 h. β-Actin served as a loading control. Numbers indicate ac-tubulin levels relative to untreated cells and normalized to β-actin expression. **(c)** Fluorescence microscopic detection of autophagosome-lysosome fusion in stable mCherry-EGFP-LC3B BE(2)-C cells cultured under 3D conditions. Graphs display ImageJ-based quantification of red or green dots/picture, respectively. Stable mCherry-EGFP-LC3B BE(2)-C cells were grown under 3D conditions for 72 h**.** Six hours before preparation for confocal microscopy, bufexamac (BUF, 30 µM) was added to the medium of the 3D cultures. Red-to-green ratio for 3D plus BUF: 1.1 +/- 0.1 (S.E.M.). **(d)** Western blot displaying LAMP-2 and SQSTM1 protein levels of cells grown in 3D culture for 72 h. Where indicated, bufexamac (BUF, 30µM) was added for the last 24 h. β-Actin served as a loading control. Numbers indicate LAMP-2 or SQSTM1 expression, respectively, relative to untreated cells and normalized to β-actin expression. **(e)** BE(2)-C cells (grown as 2D and 3D culture for four days) were treated with chloroquine (25 µM, 72 h) and vincristine (10 ng/ml, 48 h), where indicated. **(f)** BE(2)-C cells (grown as 2D and 3D culture for four days) were treated with chloroquine (25 µM, 72h) and doxorubicin (0.5 µg/ml, 48 h), where indicated. **(g)** BE(2)-C cells (grown as 2D and 3D culture for four days) were treated with bafilomaycin (BAF, 10 nM, 72h) and doxorubibcin (0.5 µg/ml, 48 h), where indicated. **(h)** Three days after transfection with ATG5, FOXO3a, HDAC6, HDAC10 or control siRNAs (siNC), BE(2)-C cells were seeded in 3D scaffolds and grown for additional 48 hours. Cells were treated with 500 ng/ml doxorubicin 24h before staining and doxorubicin fluorescence was quantified by FACS. **(a, e-g)** Relative cell number, meaning viable cells/ml normalized to solvent treated control cells, was determined with an automated cell counter. Bars represent mean values, error bars represent S.E.M. Significant differences between groups were tested using an unpaired, two-tailed t-test. *P < 0.05; **P < 0.01; ***P < 0.001; n.s. not significant.
