# Supplementary figures and images for: Three-dimensional tumor cell growth stimulates autophagic flux and recapitulates chemotherapy resistance
Source: Cell Death Dis. 2017 Aug 24;8(8):e3013–. doi: 10.1038/cddis.2017.398 (PMC5596581; doi:10.1038/cddis.2017.398)

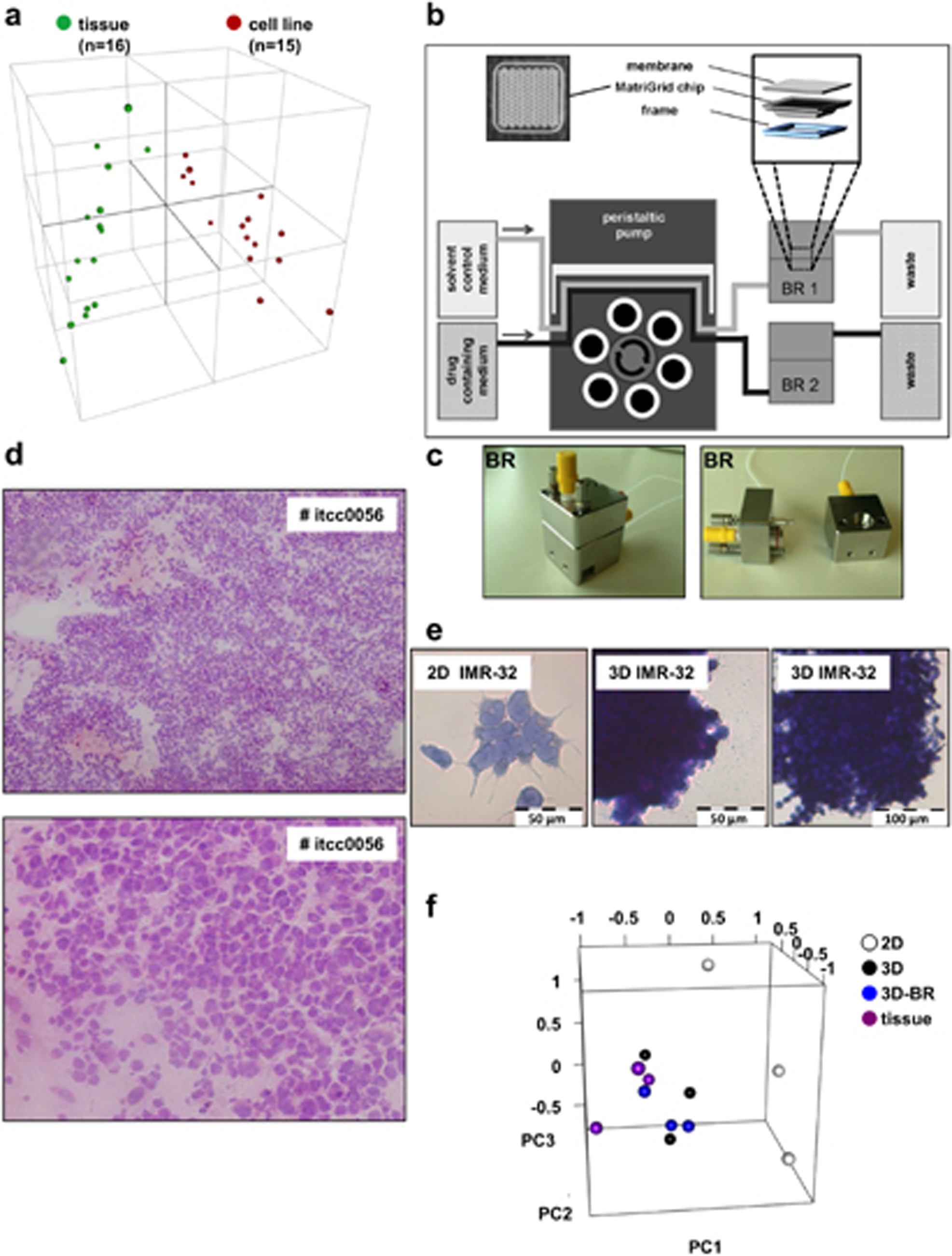

Supplement: Supplementary Figure S1 [file cddis2017398x2.tif]

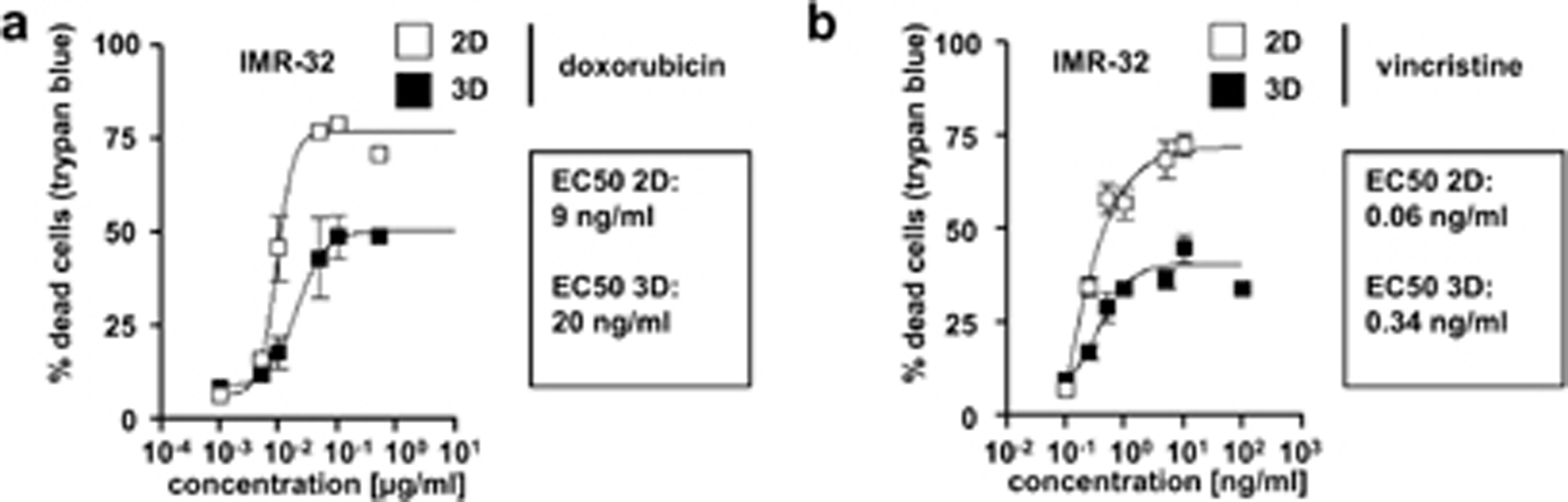

Supplement: Supplementary Figure S2 [file cddis2017398x3.tif]

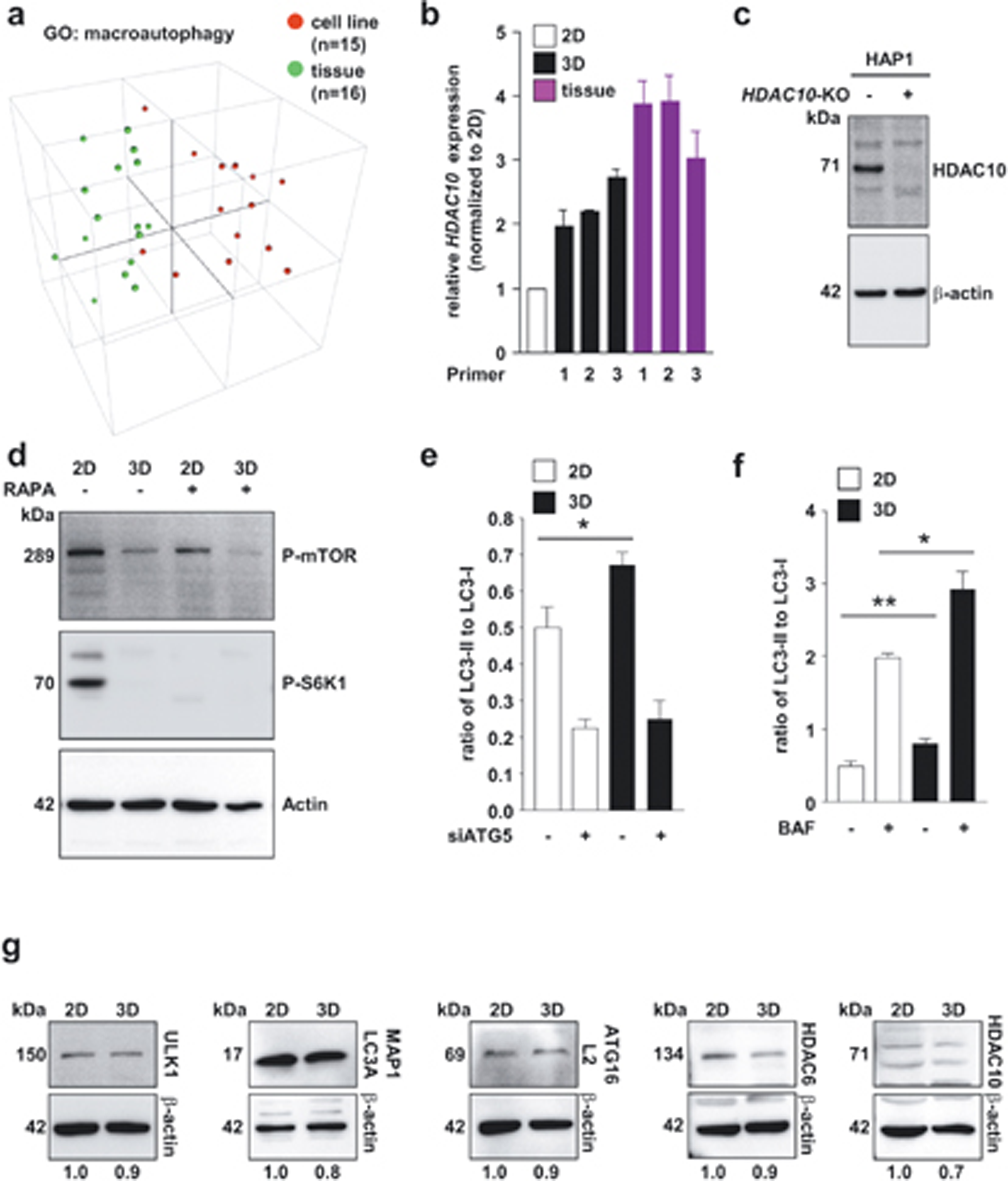

Supplement: Supplementary Figure S3 [file cddis2017398x4.tif]

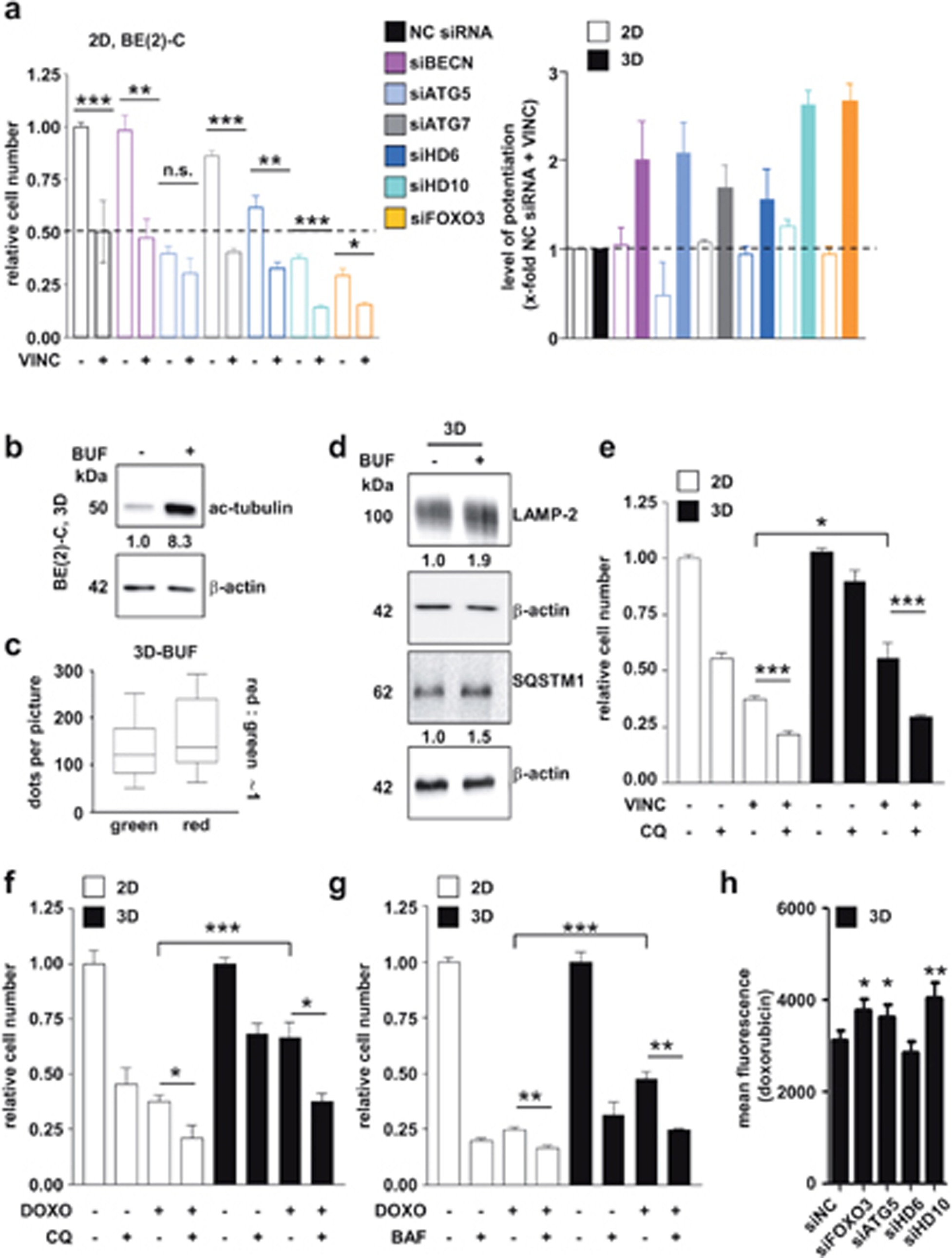

Supplement: Supplementary Figure S4 [file cddis2017398x5.tif]
