# Supplementary material for: Three-dimensional tumor cell growth stimulates autophagic flux and recapitulates chemotherapy resistance
Source: Cell Death Dis. 2017 Aug 24;8(8):e3013–. doi: 10.1038/cddis.2017.398 (PMC5596581; doi:10.1038/cddis.2017.398)
Supplement: Supplementary Table 1 [file cddis2017398x6.docx]

Table 1

**Comparison of 2D cells grown on normal to 2D cells grown on collagen-coated dishes**

|  | **2D**  **(BE(2)-C)** | **2D plus collagen (BE(2)-C)** |
| --- | --- | --- |
| **doubling time (h +/- SD)**  (0h-72h) | 27.7 +/- 1.3 | 26.5 +/- 0.8 |
| **viability untreated (% +/- SD)**  (72h) | 87.0 +/- 3.9 | 86.9 +/- 3.4 |
| **cell number (viable cells/ml)**  untreated | 779788 (=100%) | 754992 (96.8 +/- 8.2%) |
| **cell number (viable cells/ml)**  0.5 µg/ml doxorubicin 48h | 183657 (23.6 +/- 4.4%) | 176770 (22.7 +/- 4.1%) |
| **cell number (viable cells/ml)**  10 ng/ml vincristine 48h | 335531 (43.0 +/- 6.6%) | 382123 (49.0 +/- 1.0%) |
| **dead cells (% +/- SD)**  0.5 µg/ml doxorubicin 48h | 24.3 +/- 9.5 | 26.3 +/- 5.5 |
| **dead cells (% +/- SD)**  10 ng/ml vincristine 48h | 24.9 +/- 6.7 | 22.7 +/- 7.0 |
