# Supplementary material for: Three-dimensional tumor cell growth stimulates autophagic flux and recapitulates chemotherapy resistance
Source: Cell Death Dis. 2017 Aug 24;8(8):e3013–. doi: 10.1038/cddis.2017.398 (PMC5596581; doi:10.1038/cddis.2017.398)
Supplement: Supplementary Table 2 [file cddis2017398x7.docx]

Suppl. Table 2

**Comparison of 2D to 3D grown cells**

|  | **doubling time (h +/- SD)**  **(0h-72h)** | **viability (% +/- SD)**  **(72h)** |
| --- | --- | --- |
| **2D BE(2)-C** | 27.7 +/- 1.3 | 87.1 +/- 4.5 |
| **3D BE(2)-C** | 41.5 +/- 7.2 | 70.0 +/- 13.9 |
| **2D IMR-32** | 21.9 +/- 1.6 | 93.3 +/- 1.7 |
| **3D IMR-32** | 29.8 +/- 1.8 | 87.7 +/- 4.0 |
