# Supplementary material for: Three-dimensional tumor cell growth stimulates autophagic flux and recapitulates chemotherapy resistance
Source: Cell Death Dis. 2017 Aug 24;8(8):e3013–. doi: 10.1038/cddis.2017.398 (PMC5596581; doi:10.1038/cddis.2017.398)
Supplement: Supplementary Table 4 [file cddis2017398x9.docx]

Suppl. Table 4

**Correlation of TOP10 genes to “autophagy” transcription factors and neuroblastoma oncogene *MYCN***

Source: R2 database Versteeg-MYCNamp, which comprises Versteeg 88, only tumors with *MYCN* amplification (n= 16) and cell lines Versteeg 24, only cell lines with *MYCN* amplification (n= 15). The last column displays expression of TOP10 genes in 3D cultures of BE(2)-C cells 6d after transfection, measured by realtime PCR .

siFOXO3: siRNA-mediated knockdown of *FOXO3* expression in 3D-grown BE(2)-C cells

| **Correlation**  **to**  **TOP10**  **genes** | ***FOXO3*** | ***HIF-1A*** | ***TFE3*** | ***MITF*** | ***NFE2L2*** | ***TFEB*** | ***MYCN*** | ***siFOXO3*** |
| --- | --- | --- | --- | --- | --- | --- | --- | --- |
| *ULK1* | n.s. | n.s. | r= 0.514  p= 0.003 | n.s. | n.s. | n.s. | n.s. | reduced p=0.02 |
| *MAPT* | r= 0.666  p< 0.001 | r= -0.442 p= 0.01 | n.s. | n.s. | r= -0.535 p= 0.002 | r= 0.376  p= 0.04 | n.s. | reduced  p=0.04 |
| *HDAC6* | r= 0.613  p< 0.001 | r= -0.458 p= 0.01 | r= 0.498  p= 0.004 | n.s. | n.s. | n.s. | n.s. | reduced  p= 0.01 |
| *PIM2* | n.s. | n.s. | n.s. | n.s. | n.s. | n.s. | n.s. | n.s. |
| *RAB24* | n.s. | n.s. | n.s. | n.s. | n.s. | n.s. | n.s. | n.s. |
| *ABL1* | r= -0.355 p= 0.05 | n.s. | n.s. | n.s. | n.s. | n.s. | n.s. | reduced  p=0.002 |
| *MAP1LC3A* | r= 0.707  p< 0.001 | n.s. | n.s. | n.s. | r= -0.410 p= 0.02 | r= 0.516  p= 0.003 | n.s. | reduced  p=0.013 |
| *ATG16L2* | r= 0.455  p= 0.01 | n.s. | n.s. | n.s. | n.s. | r= 0.456  p= 0.01 | n.s. | reduced  p=0.0351 |
| *ATG3* | r= -0.723 p< 0.001 | n.s. | n.s. | n.s. | r= 0.450  p= 0.01 | n.s. | n.s. | increased  p=0.0153 |
| *NPC1* | r= 0.363  p= 0.04 | n.s. | r= 0.457 p= 0.01 | n.s. | n.s. | r= 0.530 p= 0.002 | n.s. | n.s. |
| *HDAC10* | r= 0.447  p= 0.01 | r= -0.363 p= 0.04 | n.s. | n.s. | r= -0.403 p= 0.02 | r= 0.434  p= 0.01 | n.s. | reduced  p= 0.02 |
