# Supplementary material for: Three-dimensional tumor cell growth stimulates autophagic flux and recapitulates chemotherapy resistance
Source: Cell Death Dis. 2017 Aug 24;8(8):e3013–. doi: 10.1038/cddis.2017.398 (PMC5596581; doi:10.1038/cddis.2017.398)
Supplement: Supplementary Table 5 [file cddis2017398x10.docx]

Suppl. Table 5

**Viability of 2D-grown BE(2)-C cells 6d after transfection measured by trypan blue staining**

| **siRNAs** | **Δ viability**^1^ | ***P*-value**^2^ |
| --- | --- | --- |
| *BECN* | 🡩 (+11.7%) | 0.0018 |
| *ATG5* | 🡩 (+8.3%) | 0.0168 |
| *ATG7* | 🡩 (+5.3%) | 0.0229 |
| *HDAC6* | 🡩 (+2.4%) | n.s. |
| *HDAC10* | **🡣** (-29.9 %) | 0.0035 |
| *FOXO3A* | 🡩 (+11,6%) | 0.0002 |

^1^ relative to negative control (NC) siRNA #1 transfected cells

^2^ one-sample t-test to test whether means of siRNA transfected samples are significantly different from 1.0 (= NC siRNA)

n.s. – not significant
